# Comparative Metabolite Profiling and Fingerprinting of Medicinal Cinnamon Bark and Its Commercial Preparations via a Multiplex Approach of GC–MS, UV, and NMR Techniques

**DOI:** 10.3390/metabo12070614

**Published:** 2022-07-01

**Authors:** Mohamed A. Farag, Sally E. Khaled, Zeina El Gingeehy, Samir Nabhan Shamma, Ahmed Zayed

**Affiliations:** 1 Pharmacognosy Department, College of Pharmacy, Cairo University, Kasr el Aini St., Cairo 11562, Egypt; 2 Pharmacognosy Department, Pharmaceutical and Drug Industries Research Institute, National Research Centre, 33 El Bohouth St., Dokki, Giza 12622, Egypt; dr.sallykhaled23@yahoo.com; 3 Chemistry Department, School of Sciences & Engineering, The American University in Cairo, New Cairo 11835, Egypt; zeinaelgingeehy@aucegypt.edu; 4 Institute of Global Health and Human Ecology, School of Sciences and Engineering, The American University in Cairo, P.O. Box 74, New Cairo 11835, Egypt; samirnabhan@aucegypt.edu; 5 Pharmacognosy Department, College of Pharmacy, Tanta University, Elguish Street (Medical Campus), Tanta 31527, Egypt; ahmed.zayed1@pharm.tanta.edu.eg

**Keywords:** cinnamon, cinnamaldehyde, chemometrics, metabolomics, SPME/GC–MS, NMR, UV/Vis

## Abstract

Various species of cinnamon (*Cinnamomum* sp.) are consumed as traditional medicine and popular spice worldwide. The current research aimed to provide the first comparative metabolomics study in nine cinnamon drugs and their different commercial preparations based on three analytical platforms, i.e., solid-phase microextraction coupled to gas chromatography–mass spectrometry method (SPME/GC–MS), nuclear magnetic resonance (NMR), and ultraviolet-visible spectrophotometry (UV/Vis) targeting its metabolome. SPME/GC–MS of cinnamon aroma compounds showed a total of 126 peaks, where (*E*)-cinnamaldehyde was the major volatile detected at 4.2–60.9% and 6.3–64.5% in authenticated and commercial preparations, respectively. Asides, modeling of the GC/MS dataset could relate the commercial products CP-1 and CP-3 to *C. cassia* attributed to their higher coumarin and low (*E*)-cinnamaldehyde content. In contrast, NMR fingerprinting identified (*E*)-methoxy cinnamaldehyde and coumarin as alternative markers for *C. verum* and *C. iners*, respectively. Additionally, quantitative NMR (qNMR) standardized cinnamon extracts based on major metabolites. UV/Vis showed to be of low discrimination power, but its orthogonal projections to latent structures discriminant analysis (OPLS-DA) S-plot showed that *C. iners* was more abundant in cinnamic acid compared to other samples. Results of this study provide potential insights into cinnamon drugs QC analysis and identify alternative markers for their discrimination.

## 1. Introduction

Spices bestow color, aroma, and taste for food, where volatile oil content accounts for such characteristics as composed of functional chemicals such as alcohols, aldehydes, amines, esters, terpenes, ketones, and others. Among the most well-characterized spices are cinnamon, ginger, turmeric, thyme, and sweet basil [1]. Particularly, cinnamon is derived from various evergreen trees and shrubs possessing aromatic characters belonging to the genus *Cinnamomum* of the laurel family (Lauraceae). It is considered among the most well-known spice that is involved in many receipts worldwide [2]. Asides from its culinary uses, it has been documented as an herbal drug in traditional tropical medicines [3], being used for the treatment of urinary tract infections, abdominal discomfort, diabetes, and muscle pain, in addition to colds and flu [2,4].

*Cinnamomum verum* (old name *C. zeylanicum*) is known as the true or Ceylon cinnamon tree native to Sri Lanka [5]. However, other *Cinnamomum* species, including *C. cassia* (Chinese cinnamon), are also consumed as possible alternatives [4], in addition to *C. burmannii* (Korintje, Java, or Indonesian cinnamon), *C. loureiroi* (Vietnamese or Saigon cinnamon), *C. tamala*, and *C. iners* [6,7]. *Cinnamomum* sp. are rich in bioactive metabolites, particularly phenylpropanoids abundant in leaf and inner bark essential oil, where eugenol and (*E*)-cinnamaldehyde are the major constituents, respectively [8]. Particularly, I-cinnamaldehyde represents 60–75% of the essential oil derived from official *C. verum* bark [5] as the most valued drug in that genus to date. Compared to *C. verum*, *C. cassia* is recognized as a lower grade owing to its richness in the toxic compound “coumarin” and lower content I(*E*)-cinnamaldehyde [9]. In terms of non-volatile bioactives, cinnamon bark contains a wide spectrum of polyphenolics such as flavonoids, proanthocyanidins, and tannins [10].

Cinnamon in modern medicine exhibit antitumor effects via dual mechanisms, i.e., upregulation of proapoptotic molecules and inhibition of NF-κ*β* and AP1 and their associated genes Bcl-2, BcL-xL, and survivin [5]. In addition, it exerts anti-diabetic effects via inhibition of *α*-amylase and *α*-glucosidase enzymes [11], recommended as flavor agents for patients with type-2 diabetes mellitus [12]. Hence, several products containing cinnamon as the major constituent are available commercially for several health benefits. Yet, the origin of these products is not always stated with no clear declaration of the cinnamon type, including exposing the consumers to potential toxicities of chemicals such as coumarin or through drug-food interactions with patients treated simultaneously with warfarin [13].

In terms of volatile organic compounds (VOCs), previous literature has commonly employed gas chromatography coupled with mass spectrometry (GC–MS) in the analysis of cinnamon essential oils extracted by steam distillation. Several VOCs were identified, where (*E*)-cinnamaldehyde, linalool, and eugenol were among the chief component by 71.5%, 7.0%, and 4.6%, respectively [14]. However, head space-solid phase micro-extraction (HS-SPME) was developed as a pre-concentration step and furtherly improved by coupling to GC–MS is a potential analytical method for aromatic herbal products [15]. Currently, solid-phase microextraction coupled to GC–MS (SPME/GC–MS) is devoted to the analysis of VOCs of low molecular weights. Asides, ultraviolet/visible (UV/Vis) spectrometry is advantageously employed as a cheap and non-destructive method for fingerprinting, albeit with its low structural identification power [15,16]. Moreover, nuclear magnetic resonance (NMR) is a powerful tool and has been recently used apart from liquid chromatography coupled to MS (LC/MS) technique for the identification of the key metabolites in different herbal crude extracts evidenced by various 1D and 2D techniques [17]. Handling the huge data generated by these platforms represents a challenge for metabolomics experts to extract valuable information aiding in sample classification, standardization, and authentication. Hence, chemometrics and multivariate data analyses (MVA) are potential candidates for such purposes, including unsupervised tools such as principal component analysis (PCA) and supervised orthogonal projections to latent structures discriminant analysis (OPLS-DA) [15]. Therefore, metabolomics-based profiling, fingerprinting, and processing of herbal products have been well documented meanwhile for quality assessment purposes, where various direct and indirect analytical platforms are used to target certain classes of phytoconstituents [18,19,20].

This study is considered a further investigation of our previous work on *Cinnamomum* sp., which aimed to reveal metabolites fingerprinting of different species based on ultra-performance liquid chromatography coupled with mass spectrometry (UPLC–MS) and gas chromatography–MS (GC–MS) following silylation and with the aid of chemometric tools [21]. This study was performed without a comparison with the metabolic profile of commercial products containing cinnamon as a principal component. It could annotate a total of 74 secondary metabolites belonging mainly to tannins, lignans, cinnamate derivatives, and flavonoids. In addition, GC–MS post derivatization addressed primary metabolites (51 metabolites) such as sugars and fatty acids. Herein, different analytical platforms were undertaken to investigate comprehensively, in a comparative and multiplex approach, the volatile and non-volatile metabolic profiles of various *Cinnamomum* sp. consumed and in its commercial preparations worldwide, including *C. cassia*, *C. verum*, *C. iners*, and *C. tamala*. VOCs were analyzed using SPME/GC–MS, while non-volatiles were monitored via NMR and UV/Visfor identification and fingerprinting of its key metabolites. Furthermore, quantitative NMR (qNMR) was employed to help quantify the major cinnamon metabolites for quality control (QC) and authentication purposes which were not possible in our previous research based on UPLC–MS and GC–MS. Considering *C. verum* economic value as a premium drug source, the geographical origin was assessed by analyzing samples from two different countries, i.e., Pakistan and Malaysia. In addition, four commercial nutraceuticals containing cinnamon as a major constituent were analyzed for the first time to determine their detailed chemical composition and aid in revealing their genotype by modeling them against authenticated specimens analyzed under the same conditions.

## 2. Results and Discussion

This study is considered a further investigation of our previous work on *Cinnamomum* sp., which was based on UPLC–MS and GC–MS following silylation and with the aid of chemometric tools. GC–MS post derivatization revealed the presence of primary metabolites, i.e., sugars, fatty acids, amino acids, and organic acids, with no focus on the aroma profile [17]. Herein, VOCs were analyzed via SPME/GC–MS, which is a rapid and sensitive technique providing a pre-concentration step that has the advantage of analyzing aroma metabolites as terpenoids such as sesquiterpenoids. In addition, the NMR platform was applied in the present study as it is considered a more reproducible analytical technique for metabolic profiling, providing the advantage of quantification of major key metabolites and facilitating quality control studies.

### 2.1. Analysis of Cinnamon Volatiles by SPME/GC–MS

SPME/GC–MS is well suited for the analysis of VOCs of aromatic plants and spices even at trace levels [22,23]. Hence, SPME/GC–MS was used in this study to discriminate between various authenticated cinnamon species of different origins along with commercial products targeting their aroma profile (Table 1).

**Figure 1:**
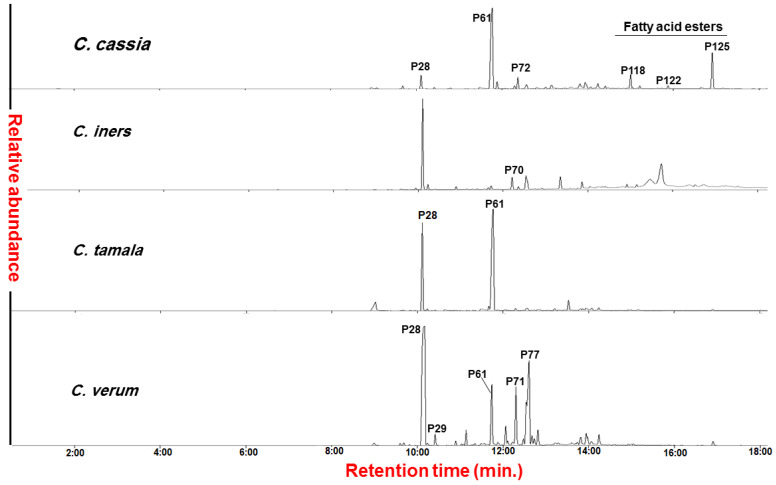
Total ion chromatograms (TIC) of investigated authenticated cinnamon samples (*Cinnamomum* sp.). The major peaks are labeled based on the tentatively identified volatiles listed in Table S1.

The SPME StableFlex (DVB/CAR/PDMS 50/30 μm) fiber was employed as it provided higher sensitivity in volatiles collection compared to the polydimethylsiloxane (PDMS) fiber and is more suitable for VOCs analysis based on our previous report [23]. Figure 1 represents the total ion chromatograms (TIC) of the four authenticated cinnamon samples, i.e., CA, CI, CT, and CV, with the labeling of the major peaks listed in Table S1.

Results showed that a total of 126 peaks were detected belonging to different chemical classes, i.e., acids (6 peaks), alcohols (24), aldehydes/ethers (21), aliphatic hydrocarbons (2), aromatic hydrocarbons (7), esters (22), ketones (6), lactones (3), oxides (3), phenols (5), pyrans/furans (4), and sesquiterpene hydrocarbons (23). Authenticated samples, including CA, showed the highest number of peaks with 122 volatiles, while CMV showed the lowest with 107 peaks. In comparison with previous studies, the current approach aided in detecting more, where Szelényi et al. reported 67 VOCs in the essential oil of *C. verum* Schaeff via SPME/GC–MS [24], only 23 compounds in *C. verum* Blume essential oil with GC–MS [14]. Likewise, only 47 compounds were identified in the n-butane extracts of different *Cinnamomum* species by GC/MS [25]. An increase in identification score may be attributed to data processing using the AMDIS program for peak deconvolution, aiding in identifying more peaks. Regarding commercial products, CP-1 showed the highest number at a total of 114 versus CP-3, with 98 peaks for commercial products.

**Figure 2:**
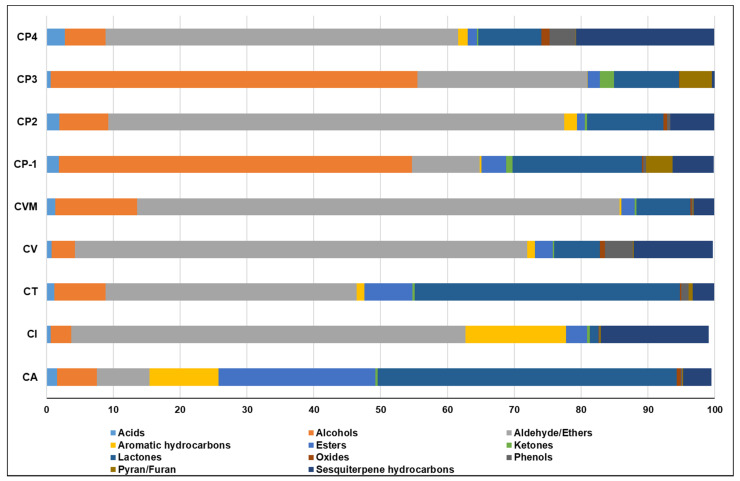
Volatile classes percentile levels in investigated cinnamon products. The sample codes are listed in Table 1.

The volatile class percentile levels in all investigated cinnamon products are depicted in Figure 2. Additionally, the major chemical classes, including the most abundant VOCs, identified using SPME/GC–MS analysis and contributed mainly to cinnamon’s unique aroma, will be discussed in the next subsections (Section 2.1.1, Section 2.1.2, Section 2.1.3, Section 2.1.4, Section 2.1.5, Section 2.1.6).

#### 2.1.1. Aldehydes/Ethers

Cinnamon bark is well known for its richness in aldehydes, i.e., (*E*)-cinnamaldehyde or also called (*E*)-cinnamaldehyde, accounting for 65–76% of its essential oil composition [3]. Chiefly, (*E*)-cinnamaldehyde contributed up to 80% of total aldehydes, to which most of the health-promoting benefits of cinnamon are attributed [13].

The results showed that aldehydes/ethers possessed the fourth place regarding the number of detected peaks, with 21 peaks after alcohols, sesquiterpene hydrocarbon, and ester. Nevertheless, they contributed with the highest relative abundance reaching up to 72% in the case of CVM. Aldehydes showed large quantitative differences as in the case of authenticated cinnamon samples accounting for 7.9% in CA versus 72.1% in CVM, and likewise in commercial products warranting that they are used for QC measures of cinnamon considering their health benefits and food value. Major aldehydes included (*E*)-cinnamaldehyde (P28), ranging from 4.2 in CA—60.9% in CVM (Table S1). The lowest content of (*E*)-cinnamaldehyde was detected in CA and in agreement with previous literature that contained 13.0–56.9 mg/g, i.e., approx. 1.3–5.7% [26]. However, [27] reported that (*E*)-cinnamaldehyde was determined at 79.4% in essential oil derived from *C. cassia* from Hexin Tang, China, suggesting a geographical origin impact.

With regards to commercial preparations, aldehyde/ether content showed large variation ranging from 10.1% to 68.2% in CP-1 and CP-2, respectively. (*E*)-Cinnamaldehyde accounted for 6.3–64.6% of the total volatile peaks, with the highest level at 64.6% in CP-2 and the lowest in CP-1 (6.3%). CP-3 also showed a lower (*E*)-cinnamaldehyde level at 22.0%, while CP-4 showed relatively higher content at ca. 48.0%, Table S1. These findings suggested that CP-1 and CP-3 were closer to that of CA, whereas CP-2 and CP-4 mimicked more that of CV/CVM based on (*E*)-cinnamaldehyde relative levels.

Next to (*E*)-cinnamaldehyde, other major aldehydes detected included *O*-methoxy cinnamaldehyde (P77), which reached 8.5 and 9.6% in CV and CVM, respectively. Benzaldehyde (P7) was also detected at 0.35–1.22% in authenticated samples, comparable with previous reports in cinnamon bark (*C. verum*) essential oil at 0.23–0.31% [28]. Interestingly, commercial products showed the opposite pattern with higher benzaldehyde levels than in authenticated samples at 0.93–2.66% versus lower *O*-methoxy cinnamaldehyde levels at (0.2–2.17%), Table S1.

Regarding ethers, eugenol (P41) was identified as the major ether in all accessions accounting for 0.02% in CVM to reach 4.7% in CI at its highest level. In contrast to cinnamon leaves, eugenol in cinnamon bark was detected at small levels and in agreement with previous reports ranging from 5.0 and 10.0% [3]. Interestingly, eugenol was reported in another study as the main VOC in *C. verum* J. Presl essential oil at 80%, while (*E*)-cinnamaldehyde reached 16.3% [29]. This oil was derived from leaves collected from Xishuangbanna Tropical Botanical Garden, China, warranting analysis from several other origins to be conclusive.

#### 2.1.2. Alcohols

Alcohols are major constituents in cinnamon bark VOCs, mainly represented by phenylethyl alcohol, isoborneol, benzyl alcohol, and *α*-terpineol. Yet, they are reported at trace levels s in the range of 0.01–0.15% [28,30]. The current study showed that alcohols amounted to the largest number of peaks, i.e., 24 peaks though detected at low levels ranging from 0.12–6.8%. The highest levels were found in CT (4.2%) and CVM (6.8%); Table S1 represented (*Z*)-3-hexen-1-ol (P6) and 3-hexen-1-ol isomer (P9) as major peaks. (*Z*)-3-Hexen-1-ol is an aroma compound that imparts a green-grassy odor of freshly cut leaves, recognized as the main fragrant in green tea [31]. However, for commercial products, including CP-1 and CP-3, alcohols accounted for 53 and 55%, respectively, represented by P6 and P9. The higher content of these alcohols might be attributed to the method of production, preparation methods, and or additional flavoring agents and odorants though not specified on the label.

Other alcohols that have been previously reported in cinnamon bark identified in this study included *α*-terpineol (P19) at 0.03–0.25% in authenticated drugs. Similarly, isoborneol (P18) was identified at 0.07–0.9% and 0.2–1.7% in authenticated and commercial products, respectively. In addition, cinnamyl alcohol (P27/P33) was detected at trace levels ranging from 0.01–1.3%. Other alcohols, i.e., linalool and borneol, were not detected in the current study though reported in the essential oil of *C. verum* J. Presl in North Brazil [32], linalool in *C. verum* [28,30], and borneol in *C. cassia* [7].

Major alcohol detected in commercial samples included cumic alcohol (P30) detected at 2.9 and 2.3% in CP-2 and CP-4, respectively, Table S1. This compound is reported to possess a spicy caraway-like odor [33] and might be added intentionally by manufacturers as a flavoring agent.

#### 2.1.3. Sesquiterpene Hydrocarbons

Likewise, in the case of alcohols, sesquiterpene hydrocarbons were reported among cinnamon bark VOCs, though at a trace level represented by *β*-caryophyllene in *C. zeylanicum* bark (1.3–6.9%) [7], in addition to calamenene, *α*-muurolene, and *γ*-muurolene [34]. The present study revealed the presence of 23 peaks amounting for 3.1–16.2% in authenticated cinnamon vs. 0.4–20.7% in commercial products of the total peak areas, Table S1.

*δ*-Cadinene was the major form in CV at 3.5%. Additionally, (*E*)-calamenene (P76) amounted to 56% of the total sesquiterpene hydrocarbons in CI detected at 9.1%, Table S1. (*E*)-Calamenene (P76) was reported at 0.5% in cinnamon bark oil prepared by Clevenger apparatus [35]. Major sesquiterpene hydrocarbons detected at higher levels in commercial products included *β*-caryophyllene (P58) found at higher levels in commercial products, i.e., CP-2 (0.4%) and CP-4 (2.5%). Likewise, *α*-muurolene (P71) and *δ*-cadinene (P75) were major constituents, especially in CP-4 at 5.6% and 6.6%, respectively.

#### 2.1.4. Esters

Esters are characterized by fine odor being responsible for the characteristic odor of several plant essential oils, though not major constituents in cinnamon oil such as (*E*)-cinnamyl acetate [3,25]. About 22 peaks belonged to esters in cinnamon specimens belonging to fatty and aromatic acid esters, Table S1. Esters accounted for 2.0–23.5% and 1.2–3.7% for authenticated drugs and commercial products, respectively. Examples of esters detected using SPME included (*E*)-cinnamyl acetate (P60, 0.02–0.9%), dimethyl phthalate (P62, 0.07–0.2%), (*E*)-ethyl cinnamate (P 63, 0.03–2.1%), and benzyl benzoate (P116, 0.1–0.9%). It is noteworthy to report that CA showed the highest levels at 23.5%, attributed to its high content of ethyl palmitate (P125) at 7.7%, detected at trace levels in other authenticated drugs, Table S1. Ethyl palmitate possesses a wax-like odor and exerts anti-inflammatory properties [36]. Whether ethyl palmitate can serve as a marker for CA should be confirmed by analyzing samples from other origins to be conclusive.

Esters showed a total abundance of 1.2–3.7% in commercial products represented by (*E*)-cinnamyl acetate (P60), dimethyl phthalate (P62), (*E*)-ethyl cinnamate (P63), and benzyl benzoate (P116) accounting for 0.03–0.2%, 0.04–0.7%, 0.01–0.1%, 0.03–0.01%, respectively, Table S1.

#### 2.1.5. Lactones

The lactone-related compounds are not a common class of VOCs except in a few plant species as in apricot [37]. However, in the current study, lactones showed large variation among authenticated drugs ranging from 1.2–44.8%, being represented by only a few numbers of peaks, including prunolide (P42), hydrocoumarin (P50), and coumarin (P61), Table S1. Higher levels were detected particularly in CA and CT samples, owing to their richness in coumarin (P61) which accounted for 44.8% and 39.6%, respectively, Table S1. The presence of a high coumarin level in cinnamon essential oil has been previously reported, especially in *C. cassia* [9], in accordance with our results, and to extend herein by identifying CT as a potential source of coumarin in cinnamon species. Coumarin is considered a toxic metabolite in high doses, where the European Food safe authority has classified cassia cinnamon among hepatotoxic and carcinogenic food. About 0.02 mg/kg/day for a 60 kg consumer is the maximum daily human exposure to coumarin from dietary sources [38]. Hence, it is recommended for consumption of cinnamon species containing low content of coumarin as CI was detected at (1.2%), CV (6.8%), and CVM (8.0%). The low level of coumarin in *C. irens* (CI) poses it as a potential flavoring agent among cinnamon species.

In comparison with previous literature, the officially used authenticated *C. verum* encompasses ca. 0.017 g/kg or 17 × 10^−4^% [39], while essential oil derived from *C. cassia* was at 0.7% [28]. Furthermore, commercial products showed comparable coumarin levels in CP-2 (11.4%) and CP-4 (9.2%), except in CP-1 at 19.1% and CP-3 at 3.3%. Interestingly, CP-3 showed the highest hydrocoumarin (P50) level detected at 6.4%, Table S1.

#### 2.1.6. Miscellaneous

Apart from the previously discussed volatile classes, other classes were identified, including phenol, ketone, pyran/furan, and oxide, though at much lower levels, Table S1. In authenticated drugs, phenols accounted for 0.12–4.2%, with the highest level detected in CVM attributed to carvacrol (P29) at 3.9%. Carvacrol was reported as a major volatile compound participating in the characteristic pungent and warm odor of cinnamon essential oil [28]. In contrast, oxide, ketone, and pyran/furan were minor classes in authenticated cinnamon, amounting for 0.08–0.8%, 0.2–0.5%, and 0.1–0.6%, respectively.

It is noteworthy to report that phenols were detected in commercial products at 0.4–3.9%, with likewise carvacrol as a major component, especially in CP-4 at 3.8%. The other classes were also identified at trace levels. Nevertheless, furfural (P5) and dihydro-3-methylene-5-methyl-2-furanone (P13) were found to be relatively abundant in CP-3 (4.7%) and CP-2 (2.9%), respectively. These products are well known to be associated with the roasting process [23,40], and whether thermal treatment was employed in the preparation of commercial products can be assessed by monitoring other markers, i.e., melanoidins, to be more conclusive.

Other (*E*)-cinnamaldehyde-related metabolites were identified in various phytochemical classes exemplified by hydrocinnamic acid (P34) and cinnamic acid (P54), detected at 0.01–1.1% and 0.02–0.3% in authenticated samples, respectively. However, they were observed at higher levels in commercial products. For instance, cinnamic acid levels reached 2.2% in CP-4, Table S1. Such an increase might confirm the hypothesis of thermal degradation revealed by the presence of furans.

### 2.2. Unsupervised Analyses of Cinnamon by SPME/GC–MS

Following the data analyses generated using SPME-GC/MS, which were discussed thoroughly in the previous sub-sections, MVDA, including unsupervised and supervised, was attempted for sample classification, marker identification, and QC of commercial products in an untargeted manner. The following subsections analyzed the dataset resulting from the GC/MS analytical platform.

Despite the clear differences observed among the VOCs profile of the investigated cinnamon samples by simple visual inspection of chromatograms, as shown in Figure 1, the dataset was subjected to unsupervised MVA, i.e., PCA, hierarchical cluster analysis (HCA), and heatmap for identified volatiles based on retention times (min).

#### 2.2.1. Analysis of the Cinnamon Authenticated Drugs and Commercial Preparations VOCs Dataset

**Figure 3:**
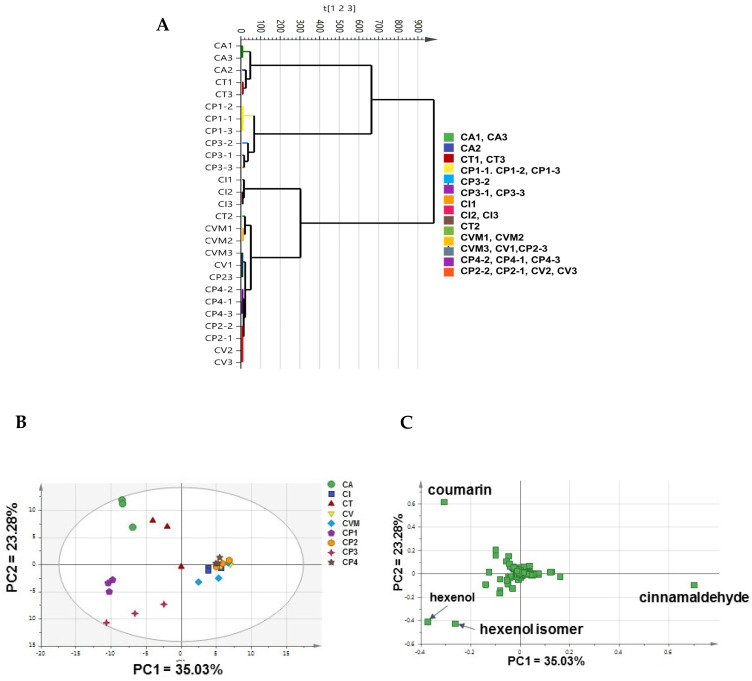
Unsupervised data analysis of the whole cinnamon dataset based on SPME-GC/MS results. (**A**) Hierarchical cluster analysis (HCA); (**B**) Principal component analysis (PCA) score plot, and (**C**) PCA loading plot. The samples code is listed in Table 1.

HCA dendrogram of the whole analyzed dataset revealed that 14 out of 27 groups were clustered mainly among two major classes. Although many biological samples were not clustered together, the CA and CT demonstrated to be clustered with CP-1 and CP-3, while CI, CV, and CVM clustered along CP-2 and CP-4 were in the second main cluster. CA and CI appeared to be unique to segregate separately as sub-clusters within the main first and second clusters, respectively, Figure 3A. These findings were in agreement with and confirmed by heatmap analysis (Supplementary Figure S1), where metabolites of low variance values were excluded.

In addition, the PCA score plot (Figure 3B) showed weak discrimination between investigated cinnamon samples, with PC1 accounting for 35.03% of the total variance, i.e., 58.31%. However, authenticated drugs CA and CT (negative side) were found to be segregated from other drugs, i.e., CV, CVM, and CI clustered on the other positive side along PC1. Moreover, CP-1 and CP-3 were also separated from CP-2 and CP-4 likewise along PC1. The overlap of CV and CVM specimens confirmed that the different geographical origin, i.e., Malaysia and Pakistan, of *C. verum* did not affect their VOCs profile and provided model validation by clustering of closely related specimens as expected.

Analysis of the PCA loading plot (Figure 3C) revealed that coumarin (P61), (*Z*)-3-hexen-1-ol (P6) and 3-hexen-1-ol isomer (P9), and cinnamaldehyde (P22) were the major discriminators to account for specimens’ segregation, with coumarin found to be related mainly to CA and CT, while hexenol to the commercial products CP-1 and CP-3. (*E*)-Cinnamaldehyde as a major component appeared enriched in CV, CVM, and CI, in addition to the commercial CP-2 and CP-4 products, and suggestive that the later products were derived from *C. verum* posing that model to predict unknown samples origin.

#### 2.2.2. Analysis of Authenticated Cinnamon Drugs VOCs Dataset

In order to confirm previous findings and aid in identifying markers for each drug, authenticated drugs dataset was analyzed separately in a new model (Supplementary Figure S2A,B). The PCA score plot (Supplementary Figure S2A) resulted in a stronger model with a total variance of 63.8%, with PC1 accounting for 47.7%. CA and CT were segregated from CV and CVM along PC1 in agreement with Figure 3B results, while CI was shown to be clustered away from CV and CVM along PC2. The geographical origin of *C. verum* was not a determinant factor in sample segregation with CV and CVM clustering together (Supplementary Figure S2A). Asides, coumarin appeared in the loading plot (Supplementary Figure S2B) to be associated with CA and CT, while (*E*)-cinnamaldehyde was correlated with CV, CVM, and CI, confirming the quantification results presented in Table S1.

These findings were consistent with those previously described in the literature regarding the high content of coumarin in *C. cassia* in comparison to *C. verum* [9]. It is noteworthy to report that the presence of coumarin as a marker of *C. tamala* is reported in the current study for the first time.

#### 2.2.3. Analysis of the Commercial Cinnamon Products VOCs Dataset

Additionally, the dataset of commercial products was analyzed separately, Supplementary Figure S2C,D. The results showed that the PCA score plot produced a stronger model with a PC1 responsible for 61.9% of the total variance (82.1%). The PCA score plot could segregate four commercial products into three clusters. The model confirmed the previous findings that CP-2 and CP-4 were highly related and could be observed to be different from CP-1 and CP-3 along PC1. Additionally, CP-1 and CP-3 could be segregated along PC2, Supplementary Figure S2C.

PCA loading plot indicated that coumarin, hexenol and hexenol isomer, and (*E*)-cinnamaldehyde were four potential markers responsible for commercial products’ segregation Supplementary Figure S2D, being more related to CP-1, CP-3, and CP-2 and CP-4, respectively. These findings were in agreement with the previous results shown in the analysis of the whole dataset (Figure 3C). Prominently, CP-1 and CP-3 used low grades of cinnamon drugs, likely *C. cassia*, in their production.

### 2.3. Supervised Analyses of Cinnamon VOCs Dataset by SPME/GC–MS

Confirming the hypothesis revealed unsupervised models of the VOCs dataset and aided in identifying biomarkers for each drug; supervised models, i.e., OPLS-DA, were further conducted since OPLS-DA has the potential to enhance specimen discrimination [23,41]. Such models functioned to verify the findings based on the effect of cinnamon inter-species and relationships to some available commercial products, as earlier revealed using PCA analysis (Figure 3B and Supplementary Figure S2A–D).

#### 2.3.1. OPLS-DA of Authenticated Cinnamon Drugs

Supervised MVA analysis for the five authenticated cinnamon drugs showed relatively strong OPLS-DA score plot model generating validation parameters of R^2^ = 92.9%, Q^2^ = 69.7%, and *p*-value = 0.0042, Supplementary Figure S3A. The model also succeeded in segregating investigated samples into four main groups confirming the previous finding, including the segregation of CV and CVM from other samples. Furthermore, CA and CI were unique species appearing as extremes at the right along PC1 and down along PC2 of the plot, respectively, Supplementary Figure S3A. Asides, (*E*)-cinnamaldehyde and *O*-methoxy cinnamaldehyde (P77) were more associated with CV and CVM, while coumarin and unknown aromatic hydrocarbon (P72) were correlated with CA in the OPLS-DA loading plot, Supplementary Figure S3B.

#### 2.3.2. OPLS-DA Analysis for Adulteration and Markers Detection

Authentication and QC of official *C. verum* via its VOCs profile were assessed using OPLS-DA, where it is usually adulterated with inferior grades consisting of other species, particularly *C. cassia* [42]. The models’ validation parameters were also calculated, including the R^2^ and Q^2^ of calibration.

Firstly, CV and CVM were modeled as one class group against CA as a second class, Supplementary Figure S4A–C. The score plot resulted in R^2^ = 99.9%, Q^2^ = 99.3%, and *p*-value = 0.0199, Supplementary Figure S4A suggestive for strong model with no over fit based on permutation plot, Supplementary Figure S4B. (*E*)-Cinnamaldehyde and coumarin were also strong determinant factors associated with CV/CVM and CA, respectively, Supplementary Figure S4C.

Secondly, CI in one class group was modeled vs. other cinnamon drugs in a second group to aid in identifying markers for CI, Supplementary Figure S4D–F. The score plot produced strong validation parameters R^2^ = 99.4%, Q^2^ = 96.9% and *p*-value = 9.31 × 10^−5,^ confirming that CI exhibited a unique aroma profile from other species Supplementary Figure S4D. Several markers appeared to be associated with such discrimination, including unknown aromatic hydrocarbons (P72 and P89), eugenol (P41), (*E*)-calamenene (P76), *α*-selinene (P70), and (*E*)-cinnamaldehyde (P28) Supplementary Figure S4F. The presence of all these markers is the first time to be identified in *C. iners* to the best of our knowledge.

### 2.4. Fingerprinting of Cinnamon NMR Dataset

**Figure 4:**
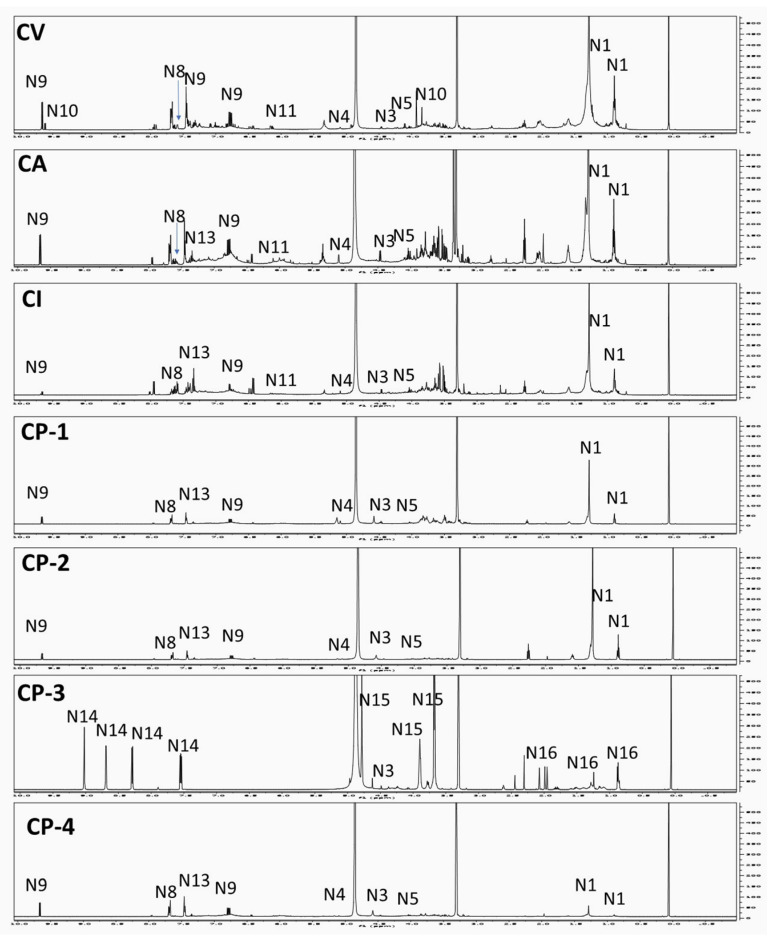
^1^H-NMR spectra of cinnamon samples showing characteristic signals for primary and secondary metabolites in spectral region (*δ*_H_ 0–10 ppm). Signal numbers correspond to those listed in Table S2 for metabolite identification using ^1^H-NMR.

NMR-based metabolomics study is recognized as a direct analytical platform and always imparts more credibility to identification results owing to its strong elucidation power aside from being quantitative (qNMR) providing a readout out of metabolite abundance in extracts [20,43]. A total of 16 metabolites were identified based on 1D- and 2D-NMR analyses. The peak assignment is summarized in Table S2, most of which were previously identified in *Cinnamomum* sp. from our previous report [44]. In the present study, ^1^H-NMR analyses were carried out to characterize and quantify major metabolites in authenticated cinnamon species, i.e., CV, CA, and CI, and commercial products. Signal assignments were further confirmed using 2D-NMR experiments, including ^1^H-^1^H COSY (correlation spectroscopy), ^1^H-^13^C HSQC (heteronuclear single quantum coherence spectroscopy), and ^1^H-^13^C HMBC (heteronuclear multiple bond correlation). Two main regions appeared in the ^1^H-NMR spectra (Figure 4): an up-field region (*δ*_H_ 0.5 to 5.5 ppm) belonging mostly to primary metabolites, namely, fatty acids (N1), glycerol (N2), and sugars (N3-N6) and a down-field region (*δ*_H_ 5.5–9.5 ppm) assigned to secondary metabolites, i.e., cinnamic acids (N7–N8), cinnamaldehydes (N9–N11), protocatechuic acid (N12) and coumarin (N13).

#### 2.4.1. Primary Metabolites

The appearance of doublets at *δ* 4.46 (*J* = 7.8 Hz), 5.09 (*J* = 3.6 Hz), and 5.14 (*J* = 3 Hz) ppm were readily assigned to anomeric protons of *β*-glucose (N3), *α*-glucose (N4), and sucrose (N6), respectively, and verified by cross-peak correlation at *δ* 98.1, 94.2 and 93.9 ppm, respectively. Moreover, the presence of doublet of doublets at *δ* 4.01 ppm (*J* = 1.2, 12.6 Hz) is characteristic of fructose (N5).

Signals characteristic for unsaturated fatty acid could be assigned in ^1^H-NMR spectra following the same identification reported in [44] assigned to ω-6 fatty acid (N1). Glycerol (N2) was readily detected from its characteristic pair of doublet of doublets at *δ* 3.5 and 3.58 ppm related to C1 and C3 methylene protons. All previous assignments were confirmed from 2D-NMR spectral data (Table S2). Primary metabolites were detected in nearly all authenticated and commercial samples except for CP-3, which showed the presence of *β*-glucose only with the absence of all other primary metabolites, Figure 4.

#### 2.4.2. Secondary Metabolites

Investigation of the aromatic region (*δ*_H_ = 6.0–9.0 ppm) revealed characteristic signals for major secondary cinnamate metabolites, namely (*Z*)- cinnamic acid (N7), (*E*)-cinnamic acid (N8), (*E*)-cinnamaldehyde (N9), cinnamaldehyde dimethyl acetal (N11) detected in nearly all samples except for CP-3, where cinnamates were totally absent, Figure 4. Identification of these secondary metabolites was on the basis of ^1^H-NMR and 2D-NMR spectral data *viz* COSY, HSQC, and HMBC (Table S2) and in accordance with those previously reported in *Cinnamomum* sp. [44]. Two additional phenolics identified in this study in cinnamon extract included (*E*)-methoxy cinnamaldehyde (N10), coumarin (N13), and protocatechuic acid (N12). (*E*)-methoxy cinnamaldehyde (N10) was identified only in *C. verum* from its three-spin system similar to that of (*E*)-cinnamaldehyde with an aldehydic doublet signal at *δ* 9.62 (*J* = 7.8 Hz) together with major singlet signal at *δ* 3.84 ppm assigned for the methoxy group. Additionally, coumarin (N13) was identified in the ^1^H-NMR spectrum down-field region via the presence of four aromatic signals (Table S2). In detail, two characteristic threefold doublet signals at *δ* 7.34 (*J* = 8.4, 7.8, 1.2 Hz) and 7.59 (*J* = 8.4, 6, 1.2 Hz) ppm were assigned for H-6 and H-7 of the benzenoid ring, respectively. Moreover, two doublets of doublets appeared at *δ* 7.4 (*J* = 6, 1.8 Hz) and 7.63 (*J* = 7.8, 1.2 Hz) ppm corresponding to H-8 and H-5, respectively. The presence of hepatotoxic coumarin indicates low-grade cinnamon and poses NMR as a new tool for its detection in cinnamon drugs. The ^1^H-NMR spectrum revealed the presence of phenolic acid with the AMX system with signals resonating at *δ* 8.01 (dd, *J* = 8.4, 1.2 Hz), 6.78 (d, *J* = 8.4 Hz) and 7.42 (overlapped) ppm assigned for H-6, H-7, and H-2 of protocatechuic acid (N12), respectively. Assignments of aromatic metabolites were verified via 2D-NMR spectral data (Table S2).

Among commercial samples, CP-3 showed the most distinct spectrum, Figure 4, assigned to three vitamins present in this preparation. Niacin or vitamin B3 (N14) was identified in the downfield region (*δ*_H_ 7.5 to 9.5 ppm), showing two downfield signals at *δ* 9.02 (d, *J* = 1.8 Hz) and 8.69 (dd, *J* = 1.2, 4.8 Hz) ppm assigned for protons at *α*- position (H-2 and H-6, respectively). Moreover, two other heteroaromatic protons resonating at *δ* 8.28 (dt, *J* = 1.8, 7.8 Hz) and 7.54 (dd, *J* = 4.8, 7.8 Hz) ppm ascribed to H-4 and H-5, respectively. Coupling constants between H-4/H-5 and H-5/H-6 ortho coupling that there are two vicinal coupling constants of 7.8 and 4.8 Hz related to different ^3^*J_HH_* between pyridine protons and assigning it as 3-monosubstituted pyridine structure of niacin [45].

Among other vitamins detected in the up-field region (*δ*_H_ 0.5 to 5.5 ppm) of CP-3 commercial products are ascorbic acid/vitamin C (N15) and *α*-tocopherol/vitamin E (N16). In detail, ascorbic acid (N15) was detected from doublet and triplet signals with *J* = 6 Hz at *δ* 3.67 and 3.89 ppm, respectively, indicating (-CH-CH_2_-) deshielded alkyl chain characteristic for ascorbic acid. The appearance of the singlet signal at *δ* 4.78 of C-4 instead of doublet indicated the instability of vitamin C and the presence of its oxidative product in CP-3. The presence of such degradation product may be due to the presence of metal ions such as manganese and chromium in CP-3 product which act as a catalyst, or may be due to storage conditions [46].

Additionally, *α*-tocopherol (N16) exhibited three singlets signals at *δ* 1.95, 1.98, and 2.07 ppm assigned to three deshielded methyl groups attached to the aromatic ring. In addition, the spectrum revealed three doublets at *δ* 0.85, 0.86, and 0.87 ppm corresponding to three methyl groups attached to the alkyl chain together with a singlet signal at *δ* 1.24 ppm ascribed to the methyl group at C-2. Finally, the presence of two signals at *δ* 1.81 (overlapped) and 2.62 (t, *J* = 6.6 Hz) ppm was assigned to H-3 and H-4, respectively. Assignments of all three vitamins were in agreement with previous NMR studies of vitamins in herbal medicines and dietary supplements [47] and posed NMR as a powerful tool to resolve multi-component herbal mixture as in the case of CP-3. The presence of these vitamins (vitamins B3, C, and E) agrees with the labeling information, considering it a multivitamin product.

#### 2.4.3. Quantification of Major Metabolites via ^1^H-NMR

^1^H-NMR was further used to determine absolute levels of identified metabolites in *Cinnamomum* samples via the integration of a single well-resolved characteristic signal in the NMR spectra. The concentration of the identified metabolites was expressed as μg/mg dry powder, which is shown in Table 2.

(*E*)-Cinnamaldehyde represented the major metabolite in all authenticated cinnamon samples ranging from 11 to 19 μg/mg. Interestingly, *Z/E*-cinnamic acid isomers were quantified in all *Cinnamomum* authenticated species at almost equal ratios showing its higher levels in CA and CI. Regarding commercial products, (*E*)-cinnamaldehyde represented the major cinnamate, followed by cinnamic acid in CP-1, CP-2, and CP-4. (*E*)-cinnamaldehyde showed its highest level at (7.3 and 6.4 μg/mg) in CP-4 and CP-1, respectively. CP-3 showed an absence of cinnamates. (*E*)-methoxy cinnamaldehyde was quantified at (11.2 μg/mg) in *C. verum* and absent in all other authenticated and commercial samples, and this is in agreement with previous results [48]. (*E*)-Cinnamaldehyde dimethyl acetal was quantified in all authenticated cinnamon samples ranging from 6.5–10.1 μg/mg) and absent in all commercial products posing it as an artifact produced during the extraction of cinnamon powder with methanol (Section 3.3.) though performed at room temperature. (*E*)-Cinnamaldehyde dimethyl acetal detection is most probably an adduct of cinnamaldehyde produced during the methanol extraction step [44]. However, the absence of such an acetal in commercial products may be attributed to the presence of cinnamaldehyde in these products at a level less than 10 μg/mg. This agrees with a 72 h stability study proving that the formation of cinnamaldehyde dimethyl acetal in methanol solution of cinnamaldehyde depends on the concentration of cinnamaldehyde and is much lower in solution with the concentration of 10 mg/g [49]. Protocatechuic acid, an antioxidant phenolic metabolite, was also detected at a comparable level (4.2 μg/mg) in *C. cassia* and *C. iners* and absent in commercial products.

Regarding sugars, contributing mainly to the palatability of cinnamon and its nutritional value, glucose (*α*- and *β*-forms) amounted to the major sugar in authenticated samples, followed by fructose. Likewise, sucrose was quantified in *C. cassia* and *C. iners* at 5.1 and 3.7 μg/mg, respectively; however, absent in *C. verum*. Glycerol, sweet sugar alcohol, was quantified at maximal level ca. 5 μg/mg in *C. cassia* and *C. iners.* While *C. cassia* showed the highest concentration of total sugars at 36.3 μg/mg, followed by *C. iners at* 24.4 μg/mg. For commercial products, monosaccharides, i.e., glucose (in its *α*- and *β*-forms) and fructose, were found major in CP-2 and CP-4, albeit sucrose was quantified at maximal level (9.0 μg/mg) in CP-1 and absent in other commercial products.

Coumarin, a hepatotoxic metabolite, reached its highest level at 13.8 and 10.4 μg/mg in CI and CA, respectively. However, the CV showed an absence of coumarin, emphasizing its genuineness. Regarding commercial products, coumarin was detected at its higher level in CP-1 at 3.2 μg/mg, while CP-2 and CP-4 at 2.7 and 3.0 μg/mg, respectively, could not be detected in CP-3. These findings are in accordance with the labeled origin being derived from *C. cassia* and confirming results revealed from VOCs profiling.

It is clear from quantitative NMR of commercial products that samples CP-1, CP-2, and CP-4 showed nearly the same pattern of metabolites with almost no significant differences (*p* ≤ 0.05) among most metabolites, except for (*E*)-cinnamaldehyde where CP-1 (6.4 μg/mg) and CP-4 (7.3 μg/mg) showed significant difference from CP-2 (4.9 μg/mg) being different from GC/MS results which showed the highest values in CP-2 and CP-4, Table S1. Such differences may be related to the extraction and analytical difference between GC/MS and NMR, where sample analysis by SPME/GC–MS undergoes a pre-concentration step followed by a chromatographic process before analysis by the MS detector. However, NMR analysis is a direct method conducted on the solvent crude extract. Additionally, NMR results could be explained by the fact that the three commercial products belong to the same origin, namely *C. cassia*, in addition to *C. burmannii* in CP-2. This could be further verified from the ratio of coumarin to (*E*)-cinnamaldehyde which is approximately (1:2) in CA together with CP-1, CP-2, and CP-4. The significant difference (*p* ≤ 0.05) in metabolites concentration (Table 2) between these three products and authenticated *C. cassia* is attributed to the presence of other excipients, i.e., cellulose and gelatin, in these commercial products, in which cinnamon powder represent less than 25% of the total powder as labeled on some products (Table 1).

Furthermore, three vitamins were detected in the commercial product CP-3 ^1^H-NMR analysis, i.e., Niacin (vitamin B3), ascorbic acid (vitamin C), *α*-tocopherol (vitamin E) quantified at 14.2, 44.5, and 5.4 μg/mg, respectively. These findings were consistent with the labeled product composition, Table 1.

#### 2.4.4. Unsupervised Analyses of Cinnamon Dataset by NMR

Authenticated (except for CT) and commercial cinnamon products were modeled. The analysis was based on the full ^1^H-NMR scale (*δ*_H_ 1–10 ppm), providing an overview of the primary metabolites that usually appear in the aliphatic shielded region and secondary bioactive metabolites appearing in the aromatic deshielded region (*δ*_H_ > 5.5 ppm).

##### Analysis of the Whole Samples’ NMR Dataset

**Figure 5:**
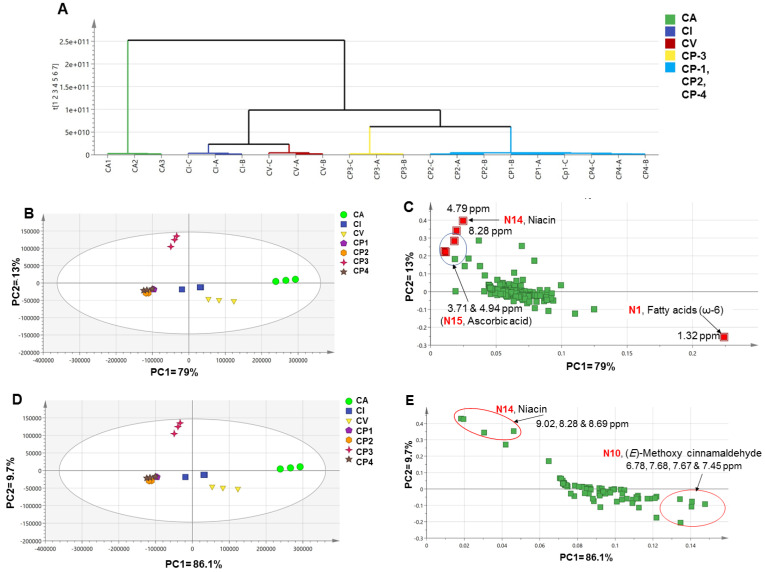
Unsupervised multivariate data analyses of the whole cinnamon dataset (except for CT) in the full ^1^H-NMR scale (*δ*_H_ 0–10 ppm) (**A**–**C**) and the aromatic region (*δ*_H_ 5.5–10 ppm) in (**D**,**E**). (**A**) Hierarchical cluster analysis (HCA), (**B**,**D**) Principal component analysis (PCA) score plot, and (**C**,**E**) PCA loading plot. The samples code is listed in Table 1. The metabolites assignment is summarized in Table S2. The red colored markers represent the samples discriminant.

Based on the full *δ*_H_ scale, HCA (Figure 5A) showed a clear clustering of CA replicates from the other cinnamon samples. Furthermore, CI and CV were likely to be similar enough to be grouped together. However, commercial products proved to be different from the authenticated samples and clustered in a separate group. Albeit, CP-3 replicates were found to be different from the other commercial products. These findings were confirmed by heatmap analysis (Supplementary Figure S5) which might be explained by the different constituents of CP-3 (Table 1). Asides, the PCA score plot showed a relatively strong model, where PC1 explained 79% of the total variance (82%) and could show segregated samples along with it. CA and CP-3 were the most unique samples confirming the HCA results, which clustered to the right-side and top-side of the model, respectively. In addition, CP-2 and CP-4 were clustered in one group proving their comparable composition, Figure 5B. Such findings were in agreement with SPME/GC–MS results, although they could not reveal the origin of commercial products. Moreover, the loading plot (Figure 5C) showed that all chemical shift markers colored red belonged to primary metabolites (*δ*_H_ < 5.0 ppm), including fatty acids and ascorbic acid at 1.32 ppm and 3.67 and 4.79 ppm, respectively. Interestingly, N1 and N15 were found to be associated with and could be recognized as markers for CA and CP-3, respectively. There was only one aromatic chemical shift at 8.28 ppm, which is related to niacin (N14) and to be a potential marker for CP-3.

The aromatic region (*δ*_H_ 5.5–10.0 ppm) is usually modeled to better identify markers from the flavor and secondary bioactive metabolites. The PCA score plot (Figure 5D) resulted in the discrimination between investigated samples in the same manner previously observed for the full scale (Figure 5B) with better discrimination parameters, i.e., PC1 = 86.1%. Asides, the PCA loading plot (Figure 5E) revealed that (*E*)-methoxy cinnamaldehyde (N10) appeared to be rich in CV and in agreement with GC/MS results being located with negative PC2 values. Furthermore, niacin (N14) was more associated with CP-3 causing its segregation on the far-left upper quadrant negative PC1 and positive PC2. However, we cannot readily predict from NMR results the origin of commercial preparations whether they belong to either CA or CV, in contrast to GC/MS findings, Figure 3.

##### Analysis of Commercial Samples’ NMR Dataset

The commercial cinnamon samples were modeled separately to show their inter-relationship based on NMR analysis. The PCA score and loading plots are shown in Supplementary Figure S6A,B, respectively. The score plot showed that also CP-3 was unique and segregated away from the other three commercial samples to the positive side of PC1, accounting for 88.3%. Moreover, the loading plot demonstrated that CP-1 and CP-4 were higher in (*E*)-cinnamaldehyde than CP-3, and in accordance with GC/MS results, Figure 3C.

#### 2.4.5. Supervised Analysis of Cinnamon NMR Dataset

Various OPLS analyses were likewise performed for the investigated cinnamon samples. Firstly, CA was modeled vs. CV (Supplementary Figure S7A,B); the model showed a distinct difference between both cinnamon samples with strong validation parameters, i.e., R^2^ = 99.2%, Q^2^ = 98.4%, *p*-value = 0.025, where (*E*)-methoxy cinnamaldehyde was found richer in the CV, which is consistent with previous findings, Table 2. Secondly, CI was plotted vs. CV and CA (Supplementary Figure S7C,D), revealing that CI was also unique from the other authenticated samples owing to its richness in coumarin (N13), compared to (*E*)-cinnamaldehyde for the authenticated samples. The validation parameters were R^2^ = 99.5%, Q^2^ = 98.8%, *p*-value = 0.0043. Finally, the commercial sample CP-3 appearing most distant in PCA analysis, as shown in Figure 5B, was investigated against the other commercial samples (Supplementary Figure S7E,F), which also showed its different compositional profile. Niacin (N14) was responsible for such difference with R^2^ = 99.4%, Q^2^ = 98.6%, *p*-value = 1.91 × 10^−5^.

### 2.5. Fingerprinting of Cinnamon Drugs Using UV/Vis

In addition to the cinnamon’s volatile constituents, it is rich in other non-volatile bioactives, including flavonoids (e.g., gossypin, hesperidin, hibifolin, and quercetin), catechin, epicatechin, and proanthocyanidins (e.g., procyanidin B2) [3,10] that cannot be easily detected using GC/MS considering their higher molecular weight or NMR attributed to the crude nature of the extract or presence at low levels [16]. These metabolites are characterized by several UV active chromophores and consequently can be used as a fingerprinting tool that could be used further as a simple, fast, and non-destructive tool for cinnamon authentication or QC analysis.

The resulted raw data from Uv/Vis spectrometer are depicted in (Supplementary Figure S8). All samples showed relatively similar spectra with mostly two λ_max_ around 225 nm and 290 nm corresponding to the phenolic bioactive content, including cinnamate derivatives, flavonoids, catechins, and proanthocyanidins [21]. However, the commercial products CP-1 and CP-3 showed different spectra, whereas CP-1 demonstrated spectra without λ_max_. In addition, CP-3 demonstrated a distinct complex UV spectrum with several λ_max_ between 245 and 270 nm, which is typical for a pyridine nucleus. These findings were consistent with the NMR results revealing the presence of niacin (N14).

#### 2.5.1. Unsupervised Multivariate Data Analyses of Cinnamon UV/Vis Dataset

Considering that the resulted UV spectra did not show characteristic features for the analyzed samples, the dataset of all investigated samples was subjected to supervised and unsupervised MVDA to aid in samples discrimination and markers identification based on characteristic λ_max_ and to further compare to modeling results derived from GC/MS dataset.

**Figure 6:**
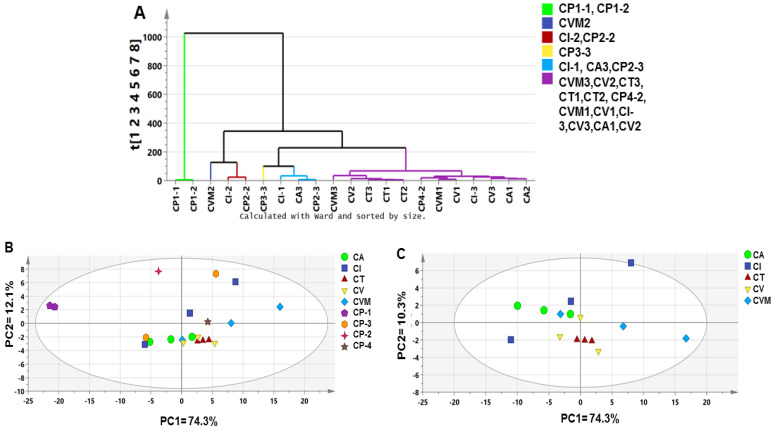
Unsupervised multivariate data analysis, including HCA (**A**) and PCA score plot (**B**) for all authenticated and commercial cinnamon products based on UV/Vis analysis, in addition to PCA score plot of authenticated cinnamon drugs (**C**). The samples code is listed in Table 1.

Compared with SPME models, HCA (Figure 6A) and PCA score plot (Figure 6B) failed to classify all samples into distinct groups, except for CP-1. CP-1 replicates were clustered away from other samples on the negative side of the PCA score plot along PC1. In general, UV models were found rather weak, with low prediction powder and failing to predict neither authenticated the sample’s geographical origin nor product origin compared to GC–MS. Nevertheless, the PCA score plot model showed a high total variance (86.4%), with PC1 accounting for 74.3%.

Similarly, modeling of authenticated samples alone showed scattered plots without a clear pattern with no segregation of biological replicates for each accession (Figure 6C). CA samples are the only clustered samples along PC2 with negative score values, though with no tight clustering of its biological replicates compared to GC/MS results (Figure 3B). Therefore, supervised MVDA, including OPLS-DA, was further attempted to extract more information from the UV dataset, failing to show clear segregation of cinnamon drugs.

#### 2.5.2. Supervised Multivariate Data Analysis of Cinnamon UV/Vis Dataset

Three models were attempted in this part focused mainly on the analysis of authenticated samples, including for all authenticated samples (Supplementary Figure S9A,B), CI against other authenticated samples (Supplementary Figure S10A–C), and finally, CA against CV and CVM (Supplementary Figure S10D–F).

Firstly, modeling of all authenticated samples showed that CI replicates positioned the most distant quarter in the score plot (Supplementary Figure S9A) far from other samples, with negative PC2 score values and in agreement with the GC–MS model, Figure 3B. Additionally, CA replicates were along the right side with CV, while CVM was positioned on the left side (Supplementary Figure S9A) and opposite to results from GC/MS showing clear segregation of CV and CVM from CA, Supplementary Figure S3. The model parameters showed weak validation parameters, i.e., R^2^ = 86.2%, Q^2^ = 60.2%, and *p*-value = 0.303, as calculated by its permutation, Supplementary Figure S9B, compared to GC/MS similar model.

Modeling of CI in one class group against other samples showed clear clustering of CI from other samples along the *x*-axis. The model resulted in strong validation parameters, i.e., R^2^ = 99.7%, Q^2^ = 97.1%, and *p*-value = 0.0026, Supplementary Figure S10A, and permutation results, Supplementary Figure S10B. The corresponding OPLS-DA line-plot was Supplementary Figure S10C revealed CI richness in compounds possessing a UV λ_max_ at 260 nm typical for cinnamic acid and confirmed their abundance in CI and in agreement with NMR results (Table 2). Such results are presented for the first time using UV/Vis analysis.

Furthermore, modeling of CA vs. CV and CVM to assess the possible adulteration of CV or CVM by the lower grade CA. OPLS-DA score plot was successful in showing a model of clearly segregated groups, Supplementary Figure S10D,E. The model also showed validation parameters of R^2^ = 99.4%, Q^2^ = 96.9%, though non-significant with *p*-value = 0.09. These findings were in agreement with that of GC/MS results (Figure 3A), confirming the authenticity of the officially used cinnamon species, *C. verum*. Additionally, the line-plot revealed that CV and CVM encompass higher proanthocyanidins and catechins than CA, attributed to the two most prominent λ_max_ at 258 and 290 nm [21], see Supplementary Figure S10F. These results are preliminary and should be confirmed using LC/MS analysis to identify exact structures.

## 3. Materials and Methods

### 3.1. Analyzed Cinnamon Samples and Origins

A total of nine cinnamon-containing products were analyzed, including five authenticated different *Cinnamomum* sp. and four commercial preparations. The details of investigated samples are summarized in Table 1. The samples included five cinnamon authenticated drugs, i.e., *C. cassia* (CA, Malaysia), *C. verum* (CVM, Malaysia), *C. iners* (CI, Malaysia), *C. tamala* (CT, Pakistan), and *C. verum* (CV, Pakistan), in addition to four commercial products containing cinnamon as major constituent. These products are in a powdered form termed Diabetruw (CP-1, 112 mg/capsule), Cinnamon Bark (GNC) (CP-2, 500 mg/capsule), Superfoods Κανέλα Extra (CP-3, 110 mg/capsule), and Spring Valley Cinnamon (CP-4, 500 mg/capsule).

### 3.2. Chemicals, Fibers, and Volatiles Analysis by SPME Coupled to GC/MS

The chemicals and reference standards were purchased from Sigma-Aldrich^®^ (St. Louis, MO, USA) unless stated otherwise. In addition, SPME StableFlex fiber coated with divinylbenzene/carboxen/polydimethylsiloxane (DVB/CAR/PDMS 50/30 μm) was purchased from Supelco^®^ (Oakville, ON, Canada). Practically, the fibers were pre-conditioned at 250 °C for 5 min based on supplier’s recommendations and then used for VOCs collection of investigated cinnamon products following the exact GC/MS protocol described previously [15,50].

### 3.3. NMR Analysis and Sample Extraction

Sample extraction followed the protocol described in [17]. Briefly, 120 mg of dried bark/commercial product powder was homogenized with 5 mL 100% methanol using a Turrax mixer (9469× *g*) for five 20 s periods, with 1 min interval to prevent heating followed by vigorous centrifuging (3000× *g* for 30 min) to remove plant debris. For analysis, four milliliters were aliquoted by a syringe, and then solvent was evaporated under nitrogen till dryness. Dried extracts were resuspended with 800 µL 100% methanol-d4 containing 0.94 mM hexamethyldisiloxane (HMDS) as an internal chemical shift NMR standard. The supernatant was centrifuged (13,000× *g* for 1 min) and transferred to a 5 mm NMR tube. All ^1^H-NMR spectra for MVA were obtained within a 48-h time interval with samples prepared instantly before data acquisition. Repeated control experiments after 48 h showed no additional variation. Three biological replicates for each specimen were extracted and analyzed in parallel under identical conditions.

### 3.4. NMR Data Acquisition

All spectra were recorded on an Agilent VNMRS 600 NMR spectrometer operating at a proton NMR frequency of 599.83 MHz using a 5 mm inverse detection cryoprobe, digital resolution 0.367 Hz/point (32 k complex data points), pulse width (pw) = 2.1 µs (30°), relaxation delay = 18 s, acquisition time = 2.0 s, number of transients = 160, and temperature = 297 K. Zero filling up to 128 K and an exponential window function with lb = 0.4 were used prior to Fourier transformation. The 2D-NMR spectra were recorded at a frequency of 599.83 MHz using standard CHEMPACK 6.2 pulse sequences (COSY, HSQC, HMBC) implemented in standard VNMRJ 4.0A spectrometer software. The HSQC experiment was optimized for ^1^*J*_CH_ = 146 Hz with DEPT-like editing and ^13^C-decoupling during acquisition time. The HMBC experiment was optimized for a long-range coupling of 8 Hz; a two-step ^1^*J*_CH_ filter was used (130–165 Hz).

### 3.5. Quantification of Major Metabolites via ^1^H-NMR

For the quantification of metabolites listed in Table S2 using NMR spectroscopy, the peak area of selected proton signals belonging to the target compounds and the peak area of the internal standard (HMDS) were integrated manually for all the samples. This protocol was carried out following the previously reported method and based on Equation 1 applied for the calculations [17].

(1) $$m_{T}{=M}_{T}\;\times \;\frac{I_{T}}{I_{\mathrm{St}}}\;\times \;\frac{x_{\mathrm{St}}}{x_{T}}{\;\times \;c}_{\mathrm{St}}{\;\times \;v}_{\mathrm{St}}$$

where m_T_: mass of the target compound in the solution used for ^1^H-NMR measurement in μg

M_T_: Molecular weight of the target compound (g/mol),
I_T_: Relative integral value of the ^1^H-NMR signal of the target compound,
I_St_: Relative integral value of the ^1^H-NMR signal of the standard compound,
X_St_: Number of protons belonging to the ^1^H-NMR signal of the standard compound,
X_T_: Number of protons belonging to the ^1^H-NMR signal of the target compound,
C_S_: Concentration of the standard compound in the solution used for ^1^H-NMR measurement (mmol/L), and
V_St_: Volume of solution used for ^1^H-NMR measurement (mL).

### 3.6. UV/Vis Analysis

Cinnamon samples, including both authenticated and commercial, were extracted by methanol (100%) following the method described by El-Hawary et al. [16]. Three replicates of authenticated and commercial cinnamon products were used in this experiment. Samples were prepared in a ratio of 1:10, with each cinnamon sample macerated with methanol (100%) for 2 h. The suspension was then centrifuged and filtered. Afterward, aliquot of each cinnamon filtrate (200 µL) was transferred to a 96-microplate well to be analyzed by the Gen 5 Greener UV microplate reader (BioTek Instruments, Inc., Winooski, VT, USA). The absorption spectra were recorded in the range of 210–600 nm.

### 3.7. Metabolites Identification, Data Processing and Multivariate Analysis

#### 3.7.1. SPME Coupled to GC/MS Analysis

Chromatographic peaks were firstly deconvoluted using AMDIS software (www.amdis.net, accessed on 18 January 2022) prior to spectral peaks matching. Additionally, relative content of each metabolite was used for data abundance extraction, being based on area normalization of each peak to total peak areas. The average response per three biological replicates was calculated per each metabolite. The identification of VOCs was based on different parameters, i.e., retention time, Kovat retention index with the aid of a series of co-chromatographed alkanes (C_8_–C_20_), spectrum matching to NIST database, and standards whenever available [15].

Untargeted MVDA, including PCA and HCA, were employed for samples dataset classification in the context of cinnamon botanical origin (*cassia* vs. *verum* vs. *tamala* vs. *iners*), genuine *C. verum* adulteration with *C. cassia* (CA vs. CV and CVM), cinnamon different commercial products (CP-1 vs. CP-2 vs. CP-3 vs. CP-4), and finally authenticated drugs vs. commercial products following same parameters described in [23]. Additionally, supervised OPLS-DA was conducted for the same dataset to aid in marker identification and confirm modeling results derived from PCA analysis. SIMCA-P version 14.0 software package (Umetrics, Umeå, Sweden) was used for modeling and all variables were mean-centered and scaled to Pareto Variance. Asides, heatmap analysis for peaks identified based on retention times (min) was conducted by MetaboAnalyst (version 5.0), choosing the option of top 25 ANOVA differential intensity.

#### 3.7.2. NMR Dataset Modeling

The NMR dataset was further processed for multivariate analysis, following the method reported by Zayed et al. [17], where the spectra were binned into buckets of a 0.04 ppm width between *δ*_H_ 11.4 to 0.4 ppm along with exclusion of signals corresponding to residual water (*δ*_H_ 5.0–4.7 ppm) and methanol (*δ*_H_ 3.4–3.25 ppm). Afterward, the data were then modeled for the full scale of chemical shift (*δ*_H_: 1–10 ppm) and aromatic region (*δ*_H_: 5.5–10 ppm) as described in GC–MS modeling. Similarly, SIMCA-P was employed for HCA and PCA modeling, while MetaboAnalyst for heatmap analysis based on chemical shifts of identified metabolites.

#### 3.7.3. UV/Vis Dataset Modeling

The spectral dataset was exported to a data matrix using excel (Excel 2010, Microsoft, Redmond, WA, USA). The matrix was constructed for all samples represented by three biological replicates. About 390 variables spanning the readings were applied. Finally, the dataset was subjected to unsupervised and supervised, including PCA and OPLS-DA, respectively, for all samples. For modeling, SIMCA-P software was applied, and all variables were mean-centered and Pareto scaled as previously described in GC–MS modeling.

## 4. Conclusions

The current study employed a comparative multiplex metabolomics-based investigation of various cinnamon bark derived from different species based on SPME-GC/MS, UV/Vis, and NMR platforms. Comparison with commercially available products was attempted to find a correlation by studying their origin and how they relate to authenticated samples. SPME/GC–MS aimed at VOCs profiling, while UV/Vis and NMR explored non-volatile constituents. SPME/GC–MS showed more valuable models regarding the origin of commercially available cinnamon products revealing that CP-1 and CP-3 were more associated with CA, while CP-2 and CP-4 were derived from higher cinnamon grade sources. These findings were attributed to the higher coumarin and low (*E*)-cinnamaldehyde contents in CP-1 and CP-3. It is observed that the presence of coumarin as a marker of *C. tamala* is reported in the current study for the first time. In addition, geographical origin appeared not to affect VOCs profile in examined cinnamon accessions. In comparison, NMR-based analyses revealed that (*E*)-methoxy cinnamaldehyde, (*E*)-cinnamaldehyde, and coumarin were richer in CV, CA, and CI, respectively. Such differences might be explained by the different applied extraction and analytical approaches. Furthermore, niacin and ascorbic acid accounted for CP-3 discrepancy in modeling results using NMR. It could be concluded from quantitative NMR that the origin of commercial products (CP-1, CP-2, and CP-4) are, to a great extent, consistent with the labeled information provided by the manufacturer, while CP-3 barely showed any cinnamon metabolites, which was inconsistent with the labeled information. Furthermore, UV/Vis showed interesting results revealing that proanthocyanidin and (*E*)-cinnamic acid were more characteristic for CV and CI, respectively. Moreover, the official CV proved its high quality, showing a unique compositional profile different from CA, confirmed by the three employed analytical platforms. Such valuable information can be applied further to other commercial products containing cinnamon in mixtures with other herbs studying the possible interferences from other phytochemical constituents. Moreover, the various potential bioactivities of cinnamon drugs, such as antioxidant and anti-microbial activities, may be related to the bioactives content revealed in this study which can be employed in future chemometric models to aid and relate compositional makeup to other effects in cinnamon drugs.

## Figures and Tables

**Table 1:** A list of cinnamon samples included in this study: botanical origin, codes, and geographical origin of authenticated drugs, in addition to trade names of commercial products.

Authenticated Cinnamon Drugs			Cinnamon Commercial Products			
Sample Code	Botanical Origin	Geographical Origin	Sample Code	Trade Name	Dose (mg)/Capsule	Cinnamon Composition
CA	*Cinamomum cassia*	Malaysia	CP-1	Diabetruw^®^	112	*C. cassia*
CVM	*Cinamomum verum*		CP-2	Cinnamon Bark (GNC)	500	*C. burmannii & C. cassia*
CI	*Cinnamomum iners*		CP-3	Superfoods Κανέλα Extra	110	*- C. cassia* cortex - Vitamin C (60 mg) - Vitamin E (13.7 mg) - Vitamin B3 (Niacin) (18.1 mg) - Vitamin B6 (1.0 mg) - Others (Zinc, Manganese, Chromium) (7.1 mg)
CT	*Cinnamomum tamala*	Pakistan	CP-4	Spring Valley Cinnamon	500	Organic cinnamon (*Cinnamomum* sp. is not stated)
CV	*Cinnamomum verum*					

**Table 2:** ^1^H-NMR quantification of major metabolites detected in different authenticated (e.g., CA, CI, and CV) and commercial (e.g., CP-1, CP-2, CP-3, and CP-4) cinnamon samples. The sample codes are listed in Table 1. Values are expressed as μg/mg dry powder ± S.D (*n* = 3), see experimental section. Chemical shifts used for metabolite quantification were determined in methanol-*d*6 and expressed as relative values to HMDS (0.94 mM final concentration). Statistical analysis is carried out by one-way ANOVA where unshared letters between groups are the significance value at *p* ≤ 0.05.

Metabolite Name	Protons Used for Quantification	Authenticated Cinnamon Products (μg/mg Dry Powder ± S.D)			Commercial Cinnamon Preparations (μg/mg Dry Powder ± S.D)			
		CV	CA	CI	CP-1	CP-2	CP-3	CP-4
Glycerol (N2)	H1/3	2.4 ± 0.1 ^b^	4.9 ± 0.2 ^a^	4.7 ± 0.5 ^a^	0.0 ^d^	0.8 ± 0.1 ^c^	0.0 ^d^	0.9 ± 0.1 ^c^
*β*-glucose (N3)	H-1	4.5 ± 0.2 ^b^	8.1 ± 0.8 ^a^	4.9 ± 0.4 ^b^	2.3 ± 0.1 ^c^	2.0 ± 0.1 ^c^	2.2 ± 0.1 ^c^	2.2 ± 0.3 ^c^
*α*-glucose (N4)	H-1	3.0 ± 0.3 ^b^	5.2 ± 0.5 ^a^	3.0 ± 0.4 ^b^	2.2 ± 0.1 ^c^	1.4 ± 0.2 ^d^	nd	1.5 ± 0.1 ^d^
Fructose (N5)	H-5	6.2 ± 0.4 ^c^	13.1 ± 0.4 ^a^	8.0 ± 0.8 ^b^	3.4 ± 0.1 ^d^	2.7 ± 0.1 ^d^	nd	3.3 ± 0.3 ^d^
Sucrose (N6)	H-1	nd	5.1 ± 0.3 ^b^	3.7 ± 0.3 ^c^	9.0 ± 0.7 ^a^	nd	nd	nd ^d^
Total sugars		16.1 ± 0.9 ^c^	36.3 ± 2.3 ^a^	24.4 ± 2.5 ^b^	16.8 ± 0.8 ^c^	6.9 ± 0.9 ^d^	2.2 ± 0.1 ^e^	7.8 ± 0.7 ^d^
(*Z*)-Cinnamic acid (N7)	H-2	4.2 ± 0.5 ^b^	7.0 ± 0.1 ^a^	6.9 ± 0.5 ^a^	2.6 ± 0.1 ^c^	2.0 ± 0.1 ^d^	nd	2.4 ± 0.1 ^c d^
(*E*)-Cinnamic acid (N8)	H-2	6.4 ± 0.6 ^b^	7.5 ± 0.2 ^a^	6.0 ± 0.6 ^b^	3.1 ± 0.2 ^c^	2.5 ± 0.1 ^d^	nd	2.8 ± 0.1 ^c d^
(*E*)-Cinnamaldehyde (N9)	H-2	13.7 ± 0.7 ^b^	19.3 ± 1.0 ^a^	11.0 ± 1.0 ^c^	6.4± 0.1 ^d^	4.9 ± 0.2 ^e^	nd	7.3 ± 0.7 ^d^
(*E*)-Methoxy cinnamaldehyde (N10)	H-2	11.2 ± 0.9 ^a^	nd	nd	nd	nd	nd	nd
Cinnamaldehyde dimethyl acetal (N11)	H-2	10.1 ± 3.1 ^a^	9.6 ± 0.3 ^a^	6.5 ± 0.7 ^b^	nd	nd	nd	nd
Total cinnamates		45.7 ± 5.8 ^a^	43.5 ± 1.4 ^a^	30.4 ± 2.7 ^b^	12.1 ± 0.2 ^c^	9.3 ± 0.3 ^c^	nd	12.5 ± 0.9 ^c^
Protocatechuic acid (N12)	H-6	2.9 ± 0.6 ^b^	4.2 ± 0.4 ^a^	4.2 ± 0.1 ^a^	nd	nd	nd	nd
Coumarin (N13)	H-5	nd	10.4 ± 0.6 ^b^	13.8 ± 1.5 ^a^	3.2 ± 0.1 ^c^	2.7 ± 0.1 ^c^	nd	3.0 ± 0.3 ^c^
Vitamin B3 (Niacin, N14)	H-2	nd	nd	nd	nd	nd	14.2 ± 1.6 ^a^	nd
Vitamin C (Ascorbic acid, N15)	H-6	nd	nd	nd	nd	nd	44.5 ± 3.4 ^a^	nd
Vitamin E (*α*-Tocopherol, N16)	C5-CH_3_	nd	nd	nd	nd	nd	5.4 ± 0.2 ^a^	nd

nd, not detected.

## Data Availability

Not applicable.

## Supplementary Materials

The following supporting information can be downloaded at: https://www.mdpi.com/article/10.3390/metabo12070614/s1, Figure S1: Heatmap analysis for all cinnamon samples based on SPME/GC–MS. The *Y*-axis represents the retention time (min) of the volatiles peak. The peak retention times for all samples and code are listed in Table S1 and Table 1, respectively. *Y*-axis to the right shows retention times in min showing relative abundance among cinnamon specimens. Figure S2: PCA score plot (A and C) and loading plot (B and D) for authenticated and commercial cinnamon samples, respectively, of cinnamon volatiles analyzed by SPME/GC–MS. The samples code is listed in Table 1, Figure S3: OPLS-DA score plot (A) and loading plot (B) of authenticated drugs for cinnamon volatiles analyzed by SPME/GC–MS. Samples code is listed in Table 1, Figure S4: OPLS-DA for authentication of *Cinnamomum verum* against other *Cinnamomum* species for volatiles analyzed by SPME/GC–MS. (A) and (D) are score plot showing validation parameters, (B) and (E) are permutation plot, and (C) and (F) are S-plot for analysis CA and CI vs. other authenticated cinnamon species. The samples code is listed in Table 1, Figure S5: Unsupervised heatmap analysis of all cinnamon dataset. (A) Full-scale (δH 0.0–10.0 ppm) and (B) aromatic region (δH 5.5–10.0 ppm). The samples code is listed in Table 1 and the metabolites assignment based on chemical shifts is summarized in Table S2. *Y*-axis to the right shows the chemical shifts showing relative abundance among cinnamon specimens. Figure S6: Unsupervised data analysis of the commercial cinnamon dataset in the aromatic region (*δ*_H_ 5.5–10.0 ppm). (A) Principal component analysis (PCA) score plot, and (B) PCA loading plot. The samples code is listed in Table 1 and the metabolites assignment is summarized in Table S2, Figure S7: Supervised data analysis (OPLS-DA) of cinnamon dataset in the aromatic region (*δ*_H_ 5.5–10.0 ppm). OPLS-DA score plot (A, C, and E) and loading plot (B, D, and F) for modelling of CA vs. CV, CI vs. CV and CA, in addition to CP3 vs. CP1, CP2 and CP4. The samples code is listed in Table 1 and the metabolites assignment is summarized in Table S2, Figure S8: UV/Vis spectra of investigated authenticated and commercial cinnamon samples. The samples code is listed in Table 1. The figure shows the unique spectrum of CP-3, Figure S9: Supervised OPLS-DA score plot (A) of authenticated cinnamon samples and permutation calculation (B). The samples code is listed in Table 1, Figure S10: Supervised OPLS-DA score plot (A) of CI against other authenticated cinnamon samples and permutation results (B), OPLS-DA line-plot (C). OPLS-DA score plot of CA against CV and CVM samples (D), permutation results (E), and OPLS-DA line-plot (F). The samples code is listed in Table 1, Table S1: The relative abundances (% ± S.E) of a list of detected peaks (126 peaks) following analysis of various cinnamon products by SPME coupled to GC/MS. The peaks were identified based on retention times, reference standards, and Kovat retention index. The samples codes are explained in Table 1, Table S2: Resonance assignments with chemical shifts (*δ*, ppm) of constituents identified in 600 MHz ^1^H, ^13^C, COSY, and HMBC NMR spectra of different authenticated (e.g., CA, CI, and CV) and commercial (e.g., CP-1, CP-2, CP-3, and CP-4) cinnamon samples. The sample codes are listed in Table 1.

## References

[1] El-Sayed S.M., Youssef A.M. (2019). Potential application of herbs and spices and their effects in functional dairy products. Heliyon.

[2] Ravindran P., Nirmal-Babu K., Shylaja M. (2003). Cinnamon and Cassia: The Genus Cinnamomum.

[3] Rao P.V., Gan S.H. (2014). Cinnamon: A multifaceted medicinal plant. Evid. Based Complementary Altern. Med..

[4] Sharifi-Rad J., Dey A., Koirala N., Shaheen S., El Omari N., Salehi B., Goloshvili T., Cirone Silva N.C., Bouyahya A., Vitalini S. (2021). *Cinnamomum* Species: Bridging Phytochemistry Knowledge, Pharmacological Properties and Toxicological Safety for Health Benefits. Front. Pharmacol..

[5] Husain I., Ahmad R., Chandra A., Raza S.T., Shukla Y., Mahdi F. (2018). Phytochemical characterization and biological activity evaluation of ethanolic extract of *Cinnamomum zeylanicum*. J. Ethnopharmacol..

[6] Chen P., Sun J., Ford P. (2014). Differentiation of the four major species of cinnamons (*C. burmannii*, *C. verum*, *C. cassia*, and *C. loureiroi*) using a flow injection mass spectrometric (FIMS) fingerprinting method. J. Agric. Food Chem..

[7] Stevens N., Allred K. (2022). Antidiabetic Potential of Volatile Cinnamon Oil: A Review and Exploration of Mechanisms Using In Silico Molecular Docking Simulations. Molecules.

[8] Zayed A., Sobeh M., Farag M.A. (2022). Dissecting dietary and semisynthetic volatile phenylpropenes: A compile of their distribution, food properties, health effects, metabolism and toxicities. Crit. Rev. Food Sci. Nutr..

[9] Wang Y.H., Avula B., Nanayakkara N.P., Zhao J., Khan I.A. (2013). Cassia cinnamon as a source of coumarin in cinnamon-flavored food and food supplements in the United States. J. Agric. Food Chem..

[10] Peng X., Ma J., Chao J., Sun Z., Chang R.C.-C., Tse I., Li E.T.S., Chen F., Wang M. (2010). Beneficial Effects of Cinnamon Proanthocyanidins on the Formation of Specific Advanced Glycation Endproducts and Methylglyoxal-Induced Impairment on Glucose Consumption. J. Agric. Food Chem..

[11] Wariyapperuma W., Kannangara S., Wijayasinghe Y.S., Subramanium S., Jayawardena B. (2020). In Vitro anti-diabetic effects and phytochemical profiling of novel varieties of *Cinnamomum zeylanicum* (L.) extracts. PeerJ.

[12] Ranasinghe P., Galappaththy P., Constantine G.R., Jayawardena R., Weeratunga H.D., Premakumara S., Katulanda P. (2017). *Cinnamomum zeylanicum* (Ceylon cinnamon) as a potential pharmaceutical agent for type-2 diabetes mellitus: Study protocol for a randomized controlled trial. Trials.

[13] Kowalska J., Tyburski J., Matysiak K., Jakubowska M., Łukaszyk J., Krzymińska J. (2021). Cinnamon as a Useful Preventive Substance for the Care of Human and Plant Health. Molecules.

[14] Kallel I., Hadrich B., Gargouri B., Chaabane A., Lassoued S., Gdoura R., Bayoudh A., Ben Messaoud E. (2019). Optimization of cinnamon (*Cinnamomum zeylanicum* Blume) essential oil extraction: Evaluation of antioxidant and antiproliferative effects. Evid. Based Complementary Altern. Med..

[15] Farag M.A., Dokalahy E.U., Eissa T.F., Kamal I.M., Zayed A. (2022). Chemometrics-Based Aroma Discrimination of 14 Egyptian Mango Fruits of Different Cultivars and Origins, and Their Response to Probiotics Analyzed via SPME Coupled to GC–MS. ACS Omega.

[16] El-Hawary E.A., Zayed A., Laub A., Modolo L.V., Wessjohann L., Farag M.A. (2022). How Does LC/MS Compare to UV in Coffee Authentication and Determination of Antioxidant Effects? Brazilian and Middle Eastern Coffee as Case Studies. Antioxidants.

[17] Zayed A., Abdelwareth A., Mohamed T.A., Fahmy H.A., Porzel A., Wessjohann L.A., Farag M.A. (2022). Dissecting coffee seeds metabolome in context of genotype, roasting degree, and blending in the Middle East using NMR and GC/MS techniques. Food Chem..

[18] Salem M.A., El-Shiekh R.A., Fernie A.R., Alseekh S., Zayed A. (2022). Metabolomics-based profiling for quality assessment and revealing the impact of drying of Turmeric (*Curcuma longa* L.). Sci. Rep..

[19] Salem M.A., Zayed A., Alseekh S., Fernie A.R., Giavalisco P. (2021). The integration of MS-based metabolomics and multivariate data analysis allows for improved quality assessment of *Zingiber officinale* Roscoe. Phytochemistry.

[20] Farag M.A., Zayed A., Sallam I.E., Abdelwareth A., Wessjohann L.A. (2022). Metabolomics-based approach for coffee beverage improvement in the context of processing, brewing methods, and quality attributes. Foods.

[21] Farag M.A., Kabbash E.M., Mediani A., Döll S., Esatbeyoglu T., Afifi S.M. (2022). Comparative metabolite fingerprinting of four different cinnamon species analyzed via UPLC-MS and GC-MS and chemometric tools. Molecules.

[22] Zouaoui N., Chenchouni H., Bouguerra A., Massouras T., Barkat M. (2020). Characterization of volatile organic compounds from six aromatic and medicinal plant species growing wild in North African drylands. NFS J..

[23] Abdelwareth A., Zayed A., Farag M.A. (2021). Chemometrics-based aroma profiling for revealing origin, roasting indices, and brewing method in coffee seeds and its commercial blends in the Middle East. Food Chem..

[24] Szelényi M.O., Erdei A.L., Jósvai J.K., Radványi D., Sümegi B., Vétek G., Molnár B.P., Kárpáti Z. (2020). Essential oil headspace volatiles prevent invasive box tree moth (*Cydalima perspectalis*) oviposition-insights from electrophysiology and behaviour. Insects.

[25] Liang Y., Li Y., Sun A., Liu X. (2019). Chemical compound identification and antibacterial activity evaluation of cinnamon extracts obtained by subcritical n-butane and ethanol extraction. Food Sci. Nutr..

[26] He Z.D., Qiao C.F., Han Q.B., Cheng C.L., Xu H.X., Jiang R.W., But P.P., Shaw P.C. (2005). Authentication and quantitative analysis on the chemical profile of cassia bark (cortex cinnamomi) by high-pressure liquid chromatography. J. Agric. Food Chem..

[27] Sun L., Zong S.-B., Li J.-C., Lv Y.-Z., Liu L.-N., Wang Z.-Z., Zhou J., Cao L., Kou J.-P., Xiao W. (2016). The essential oil from the twigs of *Cinnamomum cassia* Presl alleviates pain and inflammation in mice. J. Ethnopharmacol..

[28] Abd El-Hack M.E., Alagawany M., Abdel-Moneim A.-M.E., Mohammed N.G., Khafaga A.F., Bin-Jumah M., Othman S.I., Allam A.A., Elnesr S.S. (2020). Cinnamon (*Cinnamomum zeylanicum*) Oil as a Potential Alternative to Antibiotics in Poultry. Antibiotics.

[29] Wang R., Wang R., Yang B. (2009). Extraction of essential oils from five cinnamon leaves and identification of their volatile compound compositions. Innov. Food Sci. Emerg. Technol..

[30] Alizadeh Behbahani B., Falah F., Lavi Arab F., Vasiee M., Tabatabaee Yazdi F. (2020). Chemical Composition and Antioxidant, Antimicrobial, and Antiproliferative Activities of *Cinnamomum zeylanicum* Bark Essential Oil. Evid. Based Complementary Altern. Med..

[31] Nie C.-n., Gao Y., Du X., Bian J.-l., Li H., Zhang X., Wang C.-m., Li S.-y. (2020). Characterization of the effect of cis-3-hexen-1-ol on green tea aroma. Sci. Rep..

[32] Farias A.P.P., Monteiro O.D.S., da Silva J.K.R., Figueiredo P.L.B., Rodrigues A.A.C., Monteiro I.N., Maia J.G.S. (2020). Chemical composition and biological activities of two chemotype-oils from *Cinnamomum verum* J. Presl growing in North Brazil. J. Food Sci. Technol..

[33] FAO Online Edition: “Specifications for Flavourings”. https://www.fao.org/food/food-safety-quality/scientific-advice/jecfa/jecfa-flav/details/en/c/814/.

[34] Wei L., Lin M., Han B., Deng X., Hou W., Liao Q., Xie Z. (2016). The Comparison of Cinnamomi Cortex and *Cinnamomum burmannii* Blume Using 1H NMR and GC-MS Combined with Multivariate Data Analysis. Food Anal. Methods.

[35] Anju R., Sunitha M.C., Nevin K.G. (2018). Cinnamon extract enhances the mitochondrial reactive oxygen species production and arrests the proliferation of human colon cancer cell line, HCT-116. J. Herbs Spices Med. Plants.

[36] Saeed N.M., El-Demerdash E., Abdel-Rahman H.M., Algandaby M.M., Al-Abbasi F.A., Abdel-Naim A.B. (2012). Anti-inflammatory activity of methyl palmitate and ethyl palmitate in different experimental rat models. Toxicol. Appl. Pharmacol..

[37] Farag M.A., Ramadan N.S., Shorbagi M., Farag N., Gad H.A. (2022). Profiling of primary metabolites and volatiles in apricot (*Prunus armeniaca* L.) seed kernels and fruits in the context of its different cultivars and soil type as analyzed using chemometric tools. Foods.

[38] Lake B.G. (1999). Coumarin metabolism, toxicity and carcinogenicity: Relevance for human risk assessment. Food Chem. Toxicol. Int. J. Publ. Br. Ind. Biol. Res. Assoc..

[39] Lončar M., Jakovljević M., Šubarić D., Pavlić M., Buzjak Služek V., Cindrić I., Molnar M. (2020). Coumarins in Food and Methods of Their Determination. Foods.

[40] Zakidou P., Plati F., Matsakidou A., Varka E.-M., Blekas G., Paraskevopoulou A. (2021). Single Origin Coffee Aroma: From Optimized Flavor Protocols and Coffee Customization to Instrumental Volatile Characterization and Chemometrics. Molecules.

[41] Worley B., Powers R. (2016). PCA as a practical indicator of OPLS-DA model reliability. Curr. Metab..

[42] Shawky E., Selim D.A. (2018). Rapid Authentication and Quality Evaluation of *Cinnamomum verum* Powder Using Near-Infrared Spectroscopy and Multivariate Analyses. Planta Med..

[43] Farag M.A., Afifi S.M., Rasheed D.M., Khattab A.R. (2021). Revealing compositional attributes of Glossostemon bruguieri Desf. root geographic origin and roasting impact via chemometric modeling of SPME-GC-MS and NMR metabolite profiles. J. Food Compos. Anal..

[44] Farag M.A., Labib R.M., Noleto C., Porzel A., Wessjohann L.A. (2018). NMR approach for the authentication of 10 cinnamon spice accessions analyzed via chemometric tools. LWT.

[45] Castellano S., Sun C., Kostelnik R. (1967). Analysis of the NMR spectrum of pyridine. J. Chem. Phys..

[46] Eiff J., Monakhova Y.B., Diehl B.W.K. (2015). Multicomponent analysis of fat- and water-soluble vitamins and auxiliary substances in multivitamin preparations by qNMR. J. Agric. Food Chem..

[47] Vaysse J., Balayssac S., Gilard V., Desoubdzanne D., Malet-Martino M., Martino R. (2010). Analysis of adulterated herbal medicines and dietary supplements marketed for weight loss by DOSY 1H-NMR. Food Addit. Contaminants. Part A Chem. Anal. Control Expo. Risk Assess..

[48] Gunawardena D., Karunaweera N., Lee S., van Der Kooy F., Harman D.G., Raju R., Bennett L., Gyengesi E., Sucher N.J., Münch G. (2015). Anti-inflammatory activity of cinnamon (*C. zeylanicum* and *C. cassia*) extracts-identification of *E*-cinnamaldehyde and o-methoxy cinnamaldehyde as the most potent bioactive compounds. Food Funct..

[49] He Y., He F., Zhang Y., Wang F., Zheng X., Dai Z., Ma S. (2021). Formation of cinnamaldehyde dimethyl acetal in methanol during analysis. J. Essent. Oil Res..

[50] Khalil M.N.A., Fekry M.I., Farag M.A. (2017). Metabolome based volatiles profiling in 13 date palm fruit varieties from Egypt via SPME GC–MS and chemometrics. Food Chem..

