# Supplementary material for: Comparative Metabolite Profiling and Fingerprinting of Medicinal Cinnamon Bark and Its Commercial Preparations via a Multiplex Approach of GC–MS, UV, and NMR Techniques
Source: Metabolites. 2022 Jul 1;12(7):614. doi: 10.3390/metabo12070614 (PMC9322727; doi:10.3390/metabo12070614)
Supplement: Supplementary file 1 [file metabolites-12-00614-s001.zip › metabolites-1788670-supplementary.pdf]

Supplementary Materials

Comparative metabolite profiling and fingerprinting of medicinal cinnamon bark and its commercial preparations *via* a multiplex approach of GC–MS, UV, and NMR techniques

Supplementary Tables

Table S1: The relative abundances (% ± S.E) of a list of detected peaks (126 peaks) following analysis of various cinnamon products by SPME coupled to GC/MS. The peaks were identified based on retention times, reference standards, and Kovat retention index. The samples codes are explained in **Table 1**.

Peak No.	RT (min)	Kovat retention index (RI)	Metabolite name	Chemical class	Relative abundance (%) ± S.E								
					Authenticated cinnamon drugs					Commercial cinnamon products			
					CA	CI	CT	CV	CVM	CP-1	CP-2	CP-3	CP-4
3	2.19	515.79	Acetic acid		0.03±0.01	0.33±0.22	0.07±0.05	0.07±0.02	0.53±0.46	1.12±0.94	0.95±0.42	0.44±0.05	0.35±0.16
8	6.81	969.4	Caproic acid		0.03±0.03	0.10±0.07	0.14±0.13	0.11±0.05	0.33±0.21	0.32±0.02	0.05±0.2	0.06±0.01	0.02±0.01
16	8.993	1173.45	Benzoic acid	Acids	0.16±0.12	0.15±0.11	0.27±0.23	0.52±0.25	0.39±0.21	0.05±0.0	0.14±0.07	0.10±0.01	0.16±0.07
34	10.653	1344.68	Hydrocinnamic acid		1.14±1.0	0.02±0.01	0.68±0.31	0.03±0.01	0.03±0.01	0.15±0.01	---*	0.01±0.0	---
54	11.488	1430.94	Cinnamic acid		0.26±0.22	0.01±0.01	0.03±0.02	0.08±0.02	0.02±0.01	0.20±0.07	0.81±0.42	0.01±0.0	2.23±0.5
57	11.538	1436.26	2-Methoxy phenylacetic acid		---	---	---	---	---	---	0.01±0.0	---	0.01±0.0
**Total acids**					**1.62**	**0.62**	**1.19**	**0.81**	**1.31**	**1.85**	**1.95**	**0.62**	**2.77**
1	1.781	474.73	Ethanol		0.32±0.05	0.08±0.07	0.28±0.27	0.02±0.01	0.21±0.19	0.82±0.62	0.04±0.0	0.44±0.04	0.11±0.07
6	5.249	821.75	(*Z*)-3-Hexen-1-ol		0.17±0.12	0.12±0.09	0.61±0.59	0.18±0.1	3.09±1.84	30.89±1.7	0.15±0.08	23.30±5.08	0.02±0.01
9	7.183	1004.67	3-Hexen-1-ol isomer		1.58±1.55	0.39±0.33	4.16±4.12	0.49±0.28	6.78±3.66	11.34±2.96	0.57±0.32	29.18±4.3	0.02±0.01
10	7.283	1014.06	Unknown		---	---	---	---	0.06±0.04	7.47±1.21	---	0.96±0.04	---
15	8.46	1123.78	Phenylethyl alcohol		0.07±0.06	0.07±0.04	0.51±0.46	0.37±0.18	0.06±0.02	0.34±0.03	0.03±0.01	0.25±0.06	0.01±0.0
18	9.069	1181.04	Isoborneol		0.80±0.73	0.07±0.06	0.31±0.29	0.33±0.16	0.36±0.22	0.33±0.03	1.68±0.95	0.20±0.04	1.41±0.66
19	9.295	1201.6	*α*-Terpineol		0.16±0.15	0.10±0.09	0.25±0.23	0.10±0.05	0.03±0.02	0.15±0.0	0.88±0.5	0.05±0.0	0.82±0.39
24	9.624	1236.85	3-Phenylpropanol		0.14±0.07	0.22±0.15	0.10±0.04	0.02±0.02	0.08±0.06	0.01±0.0	0.04±0.02	0.05±0.01	0.02±0.01
27	10.064	1283.12	Cinnamyl alcohol isomer		0.03±0.01	0.27±0.1	0.06±0.02	0.04±0.04	0.07±0.03	0.29±0.02	0.01±0.0	0.01±0.0	0.02±0.02
30	10.266	1304.88	Cumic alcohol		0.21±0.2	0.01±0.0	0.19±0.17	0.02±0.01	0.03±0.01	0.11±0.05	2.85±1.6	0.03±0.01	2.25±0.93
33	10.413	1320.03±	Cinnamyl alcohol		0.30±0.02	1.25±0.69	0.09±0.01	0.75±0.12	0.7±029	0.69±0.03	0.08±0.02	0.33±0.07	0.06±0.0
86	13.202	1597.09	Caryophyllenyl alcohol		0.23±0.01	0.06±0.00	0.31±0.06	0.17±0.02	0.07±0.03	0.01±0.0	0.21±0.02	0.01±0.0	0.20±0.03
88	13.283	1605.64	Axenol	Alcohols	0.2±0.04	0.04±0.0	0.05±0.01	0.17±0.02	0.06±0.02	0.02±0.0	0.11±0.01	0.01±0.0	0.32±0.04
90	13.525	1631.82	Carotol		0.02±0.0	0.01±0.0	0.24±0.05	0.03±0.0	0.01±0.0	0.01±0.0	0.01±0.0	---	0.02±0.0
97	13.787	1661.03	Globulol		0.11±0.03	0.17±0.08	0.03±0.0	0.01±0.0	0.05±0.01	0.04±0.01	0.01±0.0	0.01±0.0	0.01±0.0
100	13.852	1668.19	Epicubenol		0.06±0.01	0.01±0.0	0.10±0.03	0.01±0.0	0.01±0.0	0.02±0.0	0.03±0.0	---	0.03±0.01
101	13.932	1677.01	*α*-Cadinol		0.66±0.12	0.06±0.0	0.16±0.04	0.45±0.05	0.34±0.09	0.04±0.01	0.34±0.01	0.01±0.01	0.46±0.08
102	13.94	1678.21	Daucol		0.47±0.03	0.04±0.0	0.06±0.02	0.15±0.02	0.12±0.03	0.03±0.0	0.12±0.01	0.01±0.0	0.14±0.03
103	14.054	1690.47	*β*-Eudesmol		0.12±0.02	0.04±0.01	0.08±0.02	0.10±0.01	0.08±0.02	0.04±0.01	0.05±0.0	0.01±0.0	0.08±0.02
104	14.069	1692.63	Cubenol isomer		0.01±0.0	0.01±0.01	0.01±0.0	0.01±0.0	0.01±0.0	0.11±0.01	---	---	0.01±0.0
106	14.111	1696.62	*β*-Bisabolol		0.03±0.01	0.02±0.02	0.01±0.0	0.02±0.0	0.02±0.01	0.03±0.01	0.01±0.0	0.01±0.0	0.01±0.0
109	14.259	1712.39	*α*-Bisabolol		0.07±0.01	0.04±0.0	0.05±0.01	0.02±0.0	0.03±0.0	0.06±0.0	0.05±0.0	0.01±0.0	0.02±0.01
112	14.466	1735.48	1-Naphthalenol		0.10±0.02	0.02±0.0	0.01±0.0	---	0.01±0.0	0.01±0.0	---	---	---
121	15.378	1834.66	Azulen-2-ol, 1,4-dimethyl-7-(1-methylethyl)-		0.04±0.01	0.01±0.01	0.01±0.0	---	---	---	---	---	---
**Total alcohols**					**5.91**	**3.12**	**7.69**	**3.45**	**12.28**	**52.85**	**7.29**	**54.89**	**6.06**

7	6.669	956.06	Benzaldehyde		0.35±0.24	1.07±0.89	1.03±0.93	0.46±0.24	1.22±0.65	1.70±0.09	1.76±0.93	2.66±0.05	0.93±0.44
11	7.671	1050.12	Salicylaldehyde		0.03±0.01	0.02±0.01	0.05±0.04	0.07±0.03	0.22±0.11	0.13±0.01	0.05±0.02	0.05±0.0	0.04±0.02
14	8.308	1109.66	Nonanal		0.02±0.0	0.05±0.02	0.02±0.0	0.01±0.0	0.54±0.28	0.04±0.01	0.01±0.0	0.04±0.01	0.01±0.0
20	9.372	1210.08	Decanal		0.03±0.02	0.03±0.01	0.02±0.0	0.01±0.0	0.07±0.03	0.05±0.01	0.02±0.01	0.04±0.01	0.01±0.0
22	9.597	1233.76	*Cis*-Cinnamaldehyde		0.02±0.01	0.29±0.15	0.11±0.04	0.80±0.3	0.22±0.07	0.03±0.0	0.43±0.18	0.07±0.02	1.23±0.32
25	9.811	1257.14	*O*-Anisaldehyde		0.45±0.36	0.03±0.02	0.11±0.04	0.15±0.07	0.04±0.02	0.10±0.02	0.13±0.07	0.03±0.0	0.01±0.0
26	9.946	1270.59	*p*-Anisaldehyde		0.05±0.03	0.45±0.35	0.15±0.02	0.03±0.0	0.04±0.01	0.04±0.01	0.02±0.0	0.03±0.0	0.01±0.01
28	10.195	1298.31	(*E*)-Cinnamaldehyde		4.16±0.47	48.51±4.85	35.09±5.28	55.63±4.34	60.88±6.3	6.31±0.74	64.6±5.98	21.93±9.81	47.94±2.55
31	10.28	1306.96	Safrole		0.02±0.01	0.05±0.02	0.05±0.02	0.10±0.04	0.06±0.01	0.13±0.07	0.08±0.02	0.03±0.0	---
36	10.734	1353.31	Piperonal	Aldehydes/ Ethers	0.34±0.2	0.02±0.01	0.03±0.01	0.01±0.0	0.01±0.0	0.06±0.01	0.06±0.03	0.03±0.01	0.09±0.07
41	10.893	1370.88	Eugenol		0.04±0.01	4.66±2.0	0.17±0.05	0.55±0.14	0.02±0.01	0.02±0.0	0.02±0.01	0.01±0.0	0.01±0.0
43	10.975	1379.1	Cerulignol		---	0.64±0.52	0.04±0.01	---	---	---	---	---	---
53	11.29	1411.28	Vanillin		0.02±0.01	0.01±0.01	0.02±0.01	0.10±0.05	0.06±0.03	0.04±0.02	0.08±0.04	0.01±0.0	0.20±0.05
73	12.454	1524.38	Acetisoeugenol		0.02±0.01	0.02±0.01	0.09±0.02	---	0.02±0.02	---	0.02±0.0	0.01±0.0	---
77	12.572	1536.24	*O*-Methoxy cinnamaldehyde		0.63±0.2	2.40±0.77	0.32±0.06	9.57±1.05	8.48±1.82	0.55±0.11	0.84±0.12	0.22±0.03	2.17±0.25
80	12.752	1553.26	*β*-Asarone		0.01±0.01	0.02±0.0	---	0.01±0.0	---	0.07±0.0	---	---	---
84	13.153	1592.48	1,5-Epoxysalvial-4(14)-ene		0.79±0.21	0.08±0.03	0.11±0.02	0.02±0.0	---	0.03±0.0	0.03±0.01	0.02±0.0	0.07±0.01
95	13.694	1651.2	Apiol		0.02±0.01	0.01±0.0	0.01±0.0	0.06±0.01	0.01±0.0	0.01±0.0	0.01±0.0	---	0.02±0.0
99	13.827	1665.6	Unknown		0.03±0.01	0.51±0.32	0.03±0.0	0.02±0.0	0.02±0.0	0.40±0.08	0.02±0.01	0.16±0.04	0.02±0.0
114	14.611	1751.89	Ylangenal		0.01±0.0	0.01±0.01	0.01±0.01	0.01±0.0	0.01±0.0	0.08±0.01	0.01±0.0	0.01±0.01	---
117	14.965	1790.56	Unknown		0.83±0.17	0.05±0.02	0.07±0.02	0.04±0.0	0.22±0.05	0.30±0.01	0.02±0.0	0.02±0.01	0.02±0.0
**Total aldehyde/ether**					**7.88**	**58.94**	**37.52**	**67.65**	**72.13**	**10.09**	**68.20**	**25.37**	**52.79**
87	13.226	1599.47	Hexadecane	Aliphatic hydrocarbons	0.34±0.12	0.73±0.22	0.05±0.01	0.18±0.02	0.03±0.01	0.12±0.01	0.02±0.0	0.03±0.0	0.01±0.0
119	15.036	1798.62	Octadecane		0.15±0.02	0.18±0.03	0.02±0.01	0.07±0.01	0.01±0.01	0.03±0.0	0.01±0.0	0.01±0.0	---
**Total aliphatic hydrocarbon**					**0.48**	**0.91**	**0.07**	**0.24**	**0.04**	**0.15**	**0.03**	**0.04**	**0.02**
49	11.157	1398.68	*β*-Vinylnaphthalene		0.02±0.0	0.76±0.25	0.01±0.01	0.01±0.0	0.01±0.01	0.02±0.01	0.01±0.0	0.01±0.0	0.01±0.0
72	12.355	1515.12	Unknown		8.84±2.89	6.81±2.63	0.09±0.02	0.03±0.01	---	0.03±0.0	0.01±0.0	---	0.19±0.16
89	13.348	1612.72	unknown	Aromatic hydrocarbons	0.09±0.05	5.95±2.45	0.29±0.11	0.04±0.01	0.02±0.01	0.05±0.03	0.01±0.0	0.01±0.0	0.01±0.01
93	13.618	1642.89	Naphthalene		0.02±0.01	---	0.01±0.0	0.02±0.0	0.01±0.0	0.01±0.0	0.01±0.0	---	0.02±0.0
96	13.725	1654.21	*α*-Corocalene		0.10±0.01	0.01±0.01	0.06±0.01	0.22±0.02	0.03±0.01	0.03±0.0	0.13±0.01	0.01±0.0	0.26±0.04
108	14.229	1709.72	Cadalene		1.03±0.24	0.10±0.04	0.36±0.1	0.77±0.1	0.15±0.03	0.13±0.0	1.75±0.24	0.02±0.01	0.88±0.17
120	15.146	1810.14	Anthracene		0.22±0.12	1.41±0.59	0.34±0.12	0.09±0.02	0.04±0.03	0.02±0.01	0.03±0.01	---	0.04±0.01
**Total aromatic hydrocarbon**					**10.33**	**15.04**	**1.15**	**1.16**	**0.28**	**0.30**	**1.96**	**0.05**	**1.41**
2	1.928	489.66	Ethyl acetate		0.80±0.77	0.16±0.15	0.28±0.28	0.24±0.1	0.20±0.19	0.24±0.04	0.19±0.0	0.99±0.44	0.28±0.14
17	9.032	1178.45	Unknown		6.55±5.68	0.13±0.05	5.44±2.55	1.18±0.34	0.33±0.07	2.01±0.4	0.12±0.04	0.11±0.02	0.21±0.07
35	10.664	1346.47	Salicylic acid methyl ether		0.40±0.21	0.02±0.01	0.16±0.07	0.01±0.0	0.04±0.03	0.06±0.02	---	0.03±0.01	---
37	10.735	1354.69	Cinnamyl formate	Esters	0.28±0.27	0.01±0.0	---	0.01±0.01	0.01±0.0	0.01±0.0	0.03±0.02	---	0.05±0.03
40	10.797	1360.5	Ethyl dihydrocinnamate		1.34±1.0	0.01±0.0	0.05±0.0	0.03±0.01	0.01±0.0	0.01±0.0	0.01±0.0	0.01±0.0	0.01±0.0
44	10.994	1381.08	Hydrocinnamyl isobutyrate		0.10±0.06	0.18±0.12	0.02±0.01	0.04±0.01	0.06±0.03	0.02±0.0	0.03±0.01	0.02±0.01	0.02±0.01

45	11.012	1383.24	Unknown		0.10±0.05	0.31±0.25	0.12±0.08	0.30±0.1	0.10±0.04	0.27±0.12	0.04±0.02	0.12±0.05	0.16±0.02
60	11.652	1446.65	Cinnamyl acetate		0.03±0.01	0.93±0.2	0.45±0.09	0.06±0.01	0.02±0.0	0.03±0.01	0.23±0.04	0.05±0.01	0.07±0.01
62	11.769	1458.04	Dimethyl phthalate		0.07±0.03	0.08±0.01	0.10±0.01	0.09±0.02	0.18±0.07	0.71±0.04	0.05±0.0	0.50±0.11	0.04±0.0
63	11.871	1467.88	(*E*)-Ethyl cinnamate		2.12±0.15	0.04±0.0	0.13±0.02	0.11±0.05	0.03±0.02	0.11±0.02	0.01±0.0	0.02±0.0	0.07±0.01
81	12.827	1561.1	Ethyl *O*-hydroxy hydrocinnamate		0.03±0.01	0.01±0.01	0.02±0.0	0.04±0.0	0.03±0.01	0.05±0.0	0.04±0.0	0.01±0.0	0.03±0.01
82	13.005	1577.51	Ethyl melilotate		0.43±0.13	0.01±0.0	0.01±0.0	---	---	0.01±0.01	0.02±0.0	---	0.02±0.01
85	13.159	1592.9	Ethyl dodecanoate		0.73±0.13	---	0.03±0.01	0.03±0.0	---	0.01±0.0	---	---	---
107	14.203	1706.76	Ethyl tridecanoate		0.26±0.03	0.01±0.0	0.02±0.0	0.02±0.0	---	---	0.01±0.0	---	0.01±0.0
115	14.839	1776.66	Ethyl 9-tetradecenoate		0.02±0.01	0.05±0.03	0.03±0.02	---	0.01±0.0	0.03±0.01	---	0.01±0.01	0.02±0.01
116	14.914	1785.1	Benzyl benzoate		0.23±0.05	0.85±0.38	0.16±0.03	0.14±0.0	0.29±0.09	0.04±0.02	0.09±0.02	0.03±0.02	0.12±0.01
118	14.991	1793.3	Ethyl myristate		1.40±0.3	0.02±0.01	0.05±0.01	0.05±0.01	---	0.01±0.0	---	---	---
122	15.871	1885.58	ethyl pentadecanoate		0.57±0.15	0.02±0.01	0.02±0.0	0.03±0.0	---	---	---	---	---
123	16.173	1917.13	Methyl palmitate		0.02±0.01	0.07±0.04	0.01±0.01	0.01±0.0	0.15±0.09	0.04±0.04	0.29±0.16	---	0.24±0.06
124	16.655	1967.6	Ethyl 9-hexadecenoate		0.18±0.06	0.22±0.13	0.01±0.01	0.01±0.0	0.54±0.34	0.02±0.02	0.01±0.0	---	0.01±0.0
125	16.889	1991.48	Ethyl palmitate		7.70±2.26	0.01±0.0	0.08±0.08	0.28±0.05	---	---	---	---	0.02±0.01
126	18.19	2123.07	Ethyl heptadecanoate		0.13±0.04	0.01±0.0	---	0.01±0.0	---	---	---	---	---
**Total esters**					**23.49**	**3.16**	**7.20**	**2.70**	**2.00**	**3.68**	**1.17**	**1.90**	**1.38**
4	2.743	571.44	Acetol	Ketones	---	0.01±0.0	---	---	0.03±0.02	0.19±0.11	0.01±0.0	1.59±0.18	---
12	7.947	1075.8	Acetophenone		0.22±0.18	0.09±0.04	0.20±0.19	0.08±0.04	0.20±0.1	0.77±0.05	0.24±0.13	0.49±0.04	0.09±0.04
39	10.794	1360.12	Piperitenone		0.01±0.01	0.02±0.01	---	0.04±0.02	0.04±0.03	---	0.02±0.01	---	0.07±0.03
92	13.58	1639.12	Acorenone B		0.01±0.0	0.22±0.09	0.01±0.0	---	---	0.01±0.0	---	---	---
105	14.075	1692.67	aR-Turmerone		0.04±0.01	0.04±0.02	0.15±0.04	0.07±0.01	0.02±0.0	0.02±0.01	0.03±0.0	0.02±0.0	0.04±0.01
110	14.262	1713.42	Mustakone.		0.03±0.01	0.09±0.08	0.01±0.0	0.02±0.0	0.02±0.0	0.02±0.0	0.02±0.0	---	0.01±0.0
**Total ketones**					**0.31**	**0.46**	**0.37**	**0.22**	**0.31**	**1.00**	**0.32**	**2.10**	**0.22**
42	10.93	1374.5	Prunolide.	Lactones	0.02±0.01	0.05±0.02	0.06±0.01	0.01±0.0	0.05±0.02	0.09±0.0	0.01±0.0	0.05±0.01	0.01±0.0
50	11.203	1402.34	Hydrocoumarin.		0.01±0.01	0.01±0.0	---	0.01±0.0	0.01±0.0	0.16±0.15	0.02±0.01	6.37±0.27	0.17±0.07
61	11.734	1454.57	Coumarin		44.76±10.74	1.19±0.41	39.56±9.47	6.76±0.65	7.97±2.34	19.11±1.0	11.41±1.95	3.30±0.04	9.22±1.81
**Total lactones**					**44.79**	**1.24**	**39.62**	**6.78**	**8.04**	**19.36**	**11.43**	**9.71**	**9.40**
68	12.107	1490.88	*Cis*-4,10-Epoxyamorphane	Oxides	0.06±0.02	---	0.06±0.01	0.17±0.04	0.02±0.01	0.01±0.0	0.10±0.03	0.02±0.0	0.10±0.01
69	12.203	1500.35	10,11-Epoxycalamenene		0.07±0.02	0.02±0.02	0.04±0.01	0.25±0.05	0.03±0.01	0.01±0.0	0.12±0.04	0.01±0.0	0.20±0.01
98	13.811	1663.51	Caryophyllene oxide		0.43±0.07	0.05±0.0	0.14±0.03	0.34±0.03	0.17±0.06	0.14±0.01	0.35±0.0	0.01±0.0	0.95±0.16
**Total oxides**					**0.55**	**0.08**	**0.24**	**0.76**	**0.23**	**0.16**	**0.58**	**0.04**	**1.25**
21	9.484	1221.7	Pyroguaiac acid	Phenols	0.01±0.0	0.01±0.01	0.01±0.0	---	0.03±0.01	0.01±0.0	0.01±0.0	0.02±0.0	0.01±0.0
29	10.211	1299.38	Carvacrol		0.01±0.0	0.05±0.03	0.74±0.71	3.90±1.95	0.02±0.01	0.23±0.18	0.02±0.01	0.01±0.0	3.76±1.87
32	10.321	1310.25	Thymol		0.05±0.02	0.04±0.02	0.18±0.05	0.19±0.11	0.01±0.0	0.08±0.0	0.29±0.13	0.01±0.0	0.01±0.0
83	13.077	1583.64	5-Hydroxycalamenene		0.03±0.0	0.01±0.01	0.06±0.01	0.02±0.0	---	0.01±0.0	0.03±0.0	---	0.06±0.01
113	14.563	1746.65	Unknown		0.15±0.04	0.01±0.01	0.08±0.02	0.08±0.01	0.07±0.01	0.07±0.01	0.07±0.01	0.01±0.0	0.09±0.02
**Total phenols**					**0.24**	**0.12**	**1.06**	**4.19**	**0.12**	**0.41**	**0.42**	**0.05**	**3.92**
5	4.953	793.12	Furfural	Pyrans/furans	0.01±0.01	0.09±0.04	0.13±0.08	0.01±0.0	0.08±0.03	1.07±0.09	0.02±0.0	4.67±0.82	0.01±0.0
13	8.025	1083.19	Dihydro-3-methylene-5-		0.02±0.02	0.02±0.02	0.46±0.41	0.14±0.07	0.04±0.02	2.86±0.34	0.02±0.01	0.12±0.02	0.01±0.0

			methyl-2-furanone										
23	9.611	1235.57	5-Hydroxymethylfurfural		0.04±0.03	0.01±0.0	0.02±0.01	---	---	0.01±0.0	0.01±0.0	---	0.01±0.01
46	11.039	1386.16	2(3*H*)-Furanone, 3-acetyldihydro-3-methyl-		0.04±0.02	0.04±0.02	---	---	0.03±0.01	0.04±0.01	---	0.03±0.0	0.01±0.0
**Total pyran/furan**					**0.11**	**0.16**	**0.61**	**0.15**	**0.16**	**3.98**	**0.05**	**4.82**	**0.03**
38	10.779	1358.17	Cadina-3,5-diene		0.11±0.07	---	---	0.01±0.01	0.01±0.0	---	---	0.01±0.0	0.01±0.0
47	11.104	1392.35	Cyclosativene.		0.39±0.36	0.040.02	0.01±0.0	0.05±0.02	0.04±0.02	0.03±0.0	0.40±0.23	0.03±0.01	0.41±0.3
48	11.13	1396.44	Copaene		0.12±0.06	0.04±0.02	0.01±0.0	0.01±0.0	0.02±0.0	0.04±0.0	---	0.05±0.01	---
51	11.259	1408.53	*β*-Elemene.		0.01±0.0	0.11±0.06	0.01±0.0	0.03±0.01	---	0.06±0.0	0.01±0.0	0.01±0.0	---
52	11.287	1411.6	(+)-Sativen		0.02±0.01	0.13±0.05	0.18±0.05	0.03±0.0	0.12±0.07	0.62±0.11	0.02±0.0	0.06±0.01	0.01±0.0
55	11.495	1431.48	Isosativene.		0.03±0.03	0.19±0.1	0.01±0.01	---	0.01±0.0	0.02±0.02	0.02±0.01	0.01±0.0	---
56	11.508	1432.57	Himachalene-1,4-diene		1.18±0.58	0.02±0.02	1.11±0.59	0.07±0.04	0.08±0.04	4.97±1.31	0.22±0.05	0.02±0.01	0.05±0.02
58	11.559	1437.46	Caryophyllene		0.01±0.0	0.09±0.06	0.02±0.01	0.10±0.03	0.04±0.02	0.01±0.0	0.37±0.18	---	2.47±0.18
59	11.631	1444.43	*trans*-*α*-Bergamotene		0.07±0.07	0.01±0.0	0.01±0.0	0.08±0.03	0.07±0.04	0.01±0.0	0.09±0.05	---	0.53±0.18
64	11.887	1469.19	Humulene		0.01±0.0	0.01±0.0	0.01±0.0	0.07±0.02	0.01±0.01	---	0.04±0.02	---	0.19±0.02
65	11.973	1477.83	4-*epi*-*α*-Acoradiene	Sesquiterpene hydrocarbons	0.03±0.01	0.01±0.0	0.01±0.0	---	---	0.01±0.0	0.09±0.03	---	0.10±0.01
66	12.042	1483.63	*γ*-Muurolene		0.14±0.05	0.11±0.06	0.10±0.03	1.08±0.66	0.34±0.2	0.02±0.01	0.82±0.31	0.01±0.0	3.33±0.16
67	12.096	1489.21	*α*-Amorphene		0.03±0.01	0.05±0.03	0.02±0.0	0.02±0.01	0.02±0.0	0.09±0.01	0.02±0.01	0.05±0.0	0.06±0.0
70	12.211	1500.79	*α*-Selinene		0.02±0.01	5.21±1.11	0.06±0.02	0.01±0.0	---	0.02±0.01	---	---	0.01±0.0
71	12.29	1508.54	*α*-Muurolene		0.62±0.18	0.04±0.01	0.54±0.13	4.94±0.65	0.75±0.42	0.02±0.0	2.68±0.78	0.02±0.0	5.59±0.48
74	12.465	1525.5	*γ*-Cadinene		0.09±0.06	0.14±0.08	0.05±0.01	0.59±0.1	0.07±0.04	0.01±0.0	0.17±0.04	0.02±0.1	0.54±0.02
75	12.533	1531.85	δ-Cadinene		0.31±0.08	0.25±0.14	0.30±0.06	3.47±0.48	1.21±0.32	0.05±0.02	1.36±0.33	0.03±0.0	6.63±0.69
76	12.551	1534.16	*trans*-Calamenene		0.19±0.12	9.07±3.35	0.60±0.23	0.08±0.02	0.05±0.02	0.10±0.05	0.02±0.0	0.03±0.01	0.02±0.02
78	12.664	1544.73	Cubenene		0.13±0.04	0.03±0.0	0.09±0.02	0.82±0.11	0.14±0.06	---	0.01±0.0	---	0.07±0.0
79	12.725	1550.73	*α*-Dehydro-ar-himachalene		0.10±0.01	0.02±0.01	0.05±0.01	0.34±0.04	0.07±0.03	0.06±0.0	0.16±0.03	0.01±0.0	0.59±0.05
91	13.563	1636.47	Clovene		0.01±0.0	0.12±0.05	0.02±0.0	---	---	0.01±0.0	---	---	---
94	13.635	1644.33	*δ*-Cadinene		0.06±0.01	0.02±0.01	0.03±0.01	0.05±0.0	0.02±0.0	0.01±0.0	0.04±0.0	---	0.06±0.01
111	14.383	1726.68	Selina-3,7(11)-diene		0.56±0.01	0.44±0.03	0.01±0.0	---	0.01±0.0	0.02±0.0	---	0.04±0.02	---
**Total sesquiterpene hydrocarbon**					**4.23**	**16.15**	**3.24**	**11.86**	**3.09**	**6.17**	**6.55**	**0.41**	**20.65**

Table S2: Resonance assignments with chemical shifts (δ, ppm) of constituents identified in 600 MHz ^1H, ^{13}C, COSY, and HMBC NMR spectra of different authenticated (e.g., CA, CI, and CV) and commercial (e.g., CP-1, CP-2, CP-3, and CP-4) cinnamon samples. The sample codes are listed in **Table 1**.

No.	Metabolite	Assignment	δ ^1H (ppm)	δ ^{13}C in HSQC (ppm)	COSY correlation δ ^1H (ppm)	HMBC correlation δ ^{13}C (ppm)	Cinnamon samples
N1	Fatty acids (ω-6)	C-1	-	177.6	-	-	CV, CA, CI, CP-1, CP-2, CP-3, CP-4
		C-2	2.27 (t, *J*=7.2 HZ)	34.8	1.59 (H-3)	C-1 (177.6), C-3 (25.9), $(CH_2)_n$ (30.2)	
		C-3	1.59 (m)	25.9	2.27 (H-2), 1.32 (CH_2)	C-1 (177.6), C-2 (34.8), $(CH_2)_n$ (30.2)	
		H-8/H-14 allylic CH_2	2.02-2.07 (m)	27.9	1.32 $(CH_2)_n$, 5.34 (olefinic Hs)	129.9 (olefinic C), 30.2 $(CH_2)_n$	
		H-11 *bis* allylic	2.77 (t, *J*= 6.6 Hz)	-	5.34 (olefinic Hs)	129.9 (olefinic C)	
		olefinic Hs	5.34 (m)	129.9	2.77 (bis allylic CH_2), 2.02-2.07 (allylic CH_2)	27.9 (allylic CH_2)	
		$(CH_2)_n$	1.32 (br. S)	30.2	0.89 (ω -1), 1.59 (H-3), 2.02-2.07 (allylic CH_2)	129.9 (olefinic C), 30.2 $(CH_2)_n$, 34.8 (C-2), 25.9 (C-3), 27.9 (allylic C), 14.3 (*t*-CH_3)	
		ω -1 (t-CH_3)	0.89 (t, *J*=7.2 Hz)	14.3	1.32 $(CH_2)_n$	23.6 (ω-2),32.9 (ω-3)	
N2	Glycerol	C-1/3	3.5 (dd, *J*= 11.4, 6 Hz)	64.3	-	64.3 (C-3), 73.7 (C-2)	CV, CA, CI, CP-2, CP-4
		C-1/3	3.58 (dd, *J*= 11.4, 4.8 Hz)	64.3	-	64.3 (C-3), 73.7 (C-2)	
		C-2	3.64 (m)	73.7	-	64.3 (C-1/3)	
N3	*β*-glucose	C-1	4.46 (d, *J*=7.8 Hz)	98.1	3.11 (H-2)	76.1 (C-2)	CV, CA, CI, CP-1, CP-2, CP-3, CP-4
		C-2	3.11 (dd, *J*= 9, 7.8 Hz)	76.1	4.46 (H-1), 3.35 (H-3)	98.1 (C-1), 78.0 (C-3)	
N4	*α*-glucose	C-1	5.09 (d, *J*=3.6 Hz)	94.2	3.34 (H-2)	73.7 (C-2)	CV, CA, CI, CP-1, CP-2, CP-4
		C-2	3.34 (dd, *J*= 3.6, 9.6 Hz)	73.7	5.09 (H-1)	-	

N5	Fructose	C-5	4.01 (dd, *J*=1.2. 12.6 Hz)	64.3	3.57 (H-4)	71.1	CV, CA, CI, CP-1, CP-2, CP-4
N6	Sucrose	C-1	5.14 (d, *J*=3 Hz)	93.9	3.4 (H-2)	74.7 (C-2)	CA, CI, CP-1
		C-2	3.4 (dd, *J*=3.6, 9.6 Hz)	74.7	5.14 (H-1), 3.8 (m)	-	
		C-3,4,6	3.8 (m)	73.7	3.4 (H-2)	-	
		C-5	3.49 (td, *J*=9, 1.8 Hz)	-	-	-	
N7	(*Z*)-Cinnamic acid	C-1	-	163.7	-	-	CV, CA, CI, CP-1, CP-2, CP-4
		C-2	6.42 (d, *J*= 9.6 Hz)	116.9	7.94 (H-3)	163.7 (C-1)	
		C-3	7.94 (d, *J*= 9.6 Hz)	145.5	6.42 (H-2)	163.7 (C-1), 116.9 (C-2),	
N8	(*E*)-Cinnamic acid	C-1	-	170.3	-	-	CV, CA, CI, CP-1, CP-2, CP-4
		C-2	6.47 (d, *J*=15.6 Hz)	119.4	7.63 (H-3)	170.3 (C-1), 146 (C-3)	
		C-3	7.63 (d, *J*=15.6 Hz)	146	6.47 (H-2)	170.3 (C-1)	
		C-4	-	133.1	-	-	
		C-5,9	7.63 (dd, *J*= 7.8, 1.2 Hz)	-	-	146 (C-3), 133.1 (C-4)	
N9	(*E*)-Cinnamaldehyde	C-1	9.67 d (J=7.8 Hz)	195.9	6.78(H-2)	129.3 (C-2)	CV, CA, CI, CP-1, CP-2, CP-4
		C-2	6.78 (dd, *J*= 15.6, 7.8 Hz)	129.3	7.68 (H-3), 9.67 (H-1)	151.9 (C-3), 134.1 (C-4)	
		C-3	7.68 (d, *J*=15.6Hz)	151.9	6.78 (H-2)	195.9 (C-1), 129.3 (C-2)	
		C-4	-	134.1	-	-	
		C-5,9	7.67 (dd, *J*= 8.4, 2.4 Hz)	129.6	7.45 (H-6,7,8)	-	
		C-7	7.45 (m)	nd	7.67 (H-5,9)	-	
		C-6,8	7.45(m)	129.9	7.67 (H-5,9)	-	
N10	(*E*)-Methoxy cinnamaldehyde	C-1	9.62 (d, *J*=7.8 Hz)	-	-	-	CV
		C-2	6.82 (dd, *J*= 16,7.8 Hz)	-	-	-	
		C-3	7.93 (d, *J*=16 Hz)	-	-	-	
		OCH_3	3.84 (s)	-	-	-	

N11	Cinnamaldehyde dimethyl acetal	C-1	4.92 (dd, *J*= 5.4, 1.2 Hz)	104.6	6.14 (H-2)	53.1 (OCH_3)	CV, CA, CI
		C-2	6.14 (dd, *J*=16.2, 5.4 Hz)	-	4.92 (H-1), 6.72 (H-3)	137.4 (C-4)	
		C-3	6.72 (d, *J*=16.2 Hz)	-	6.14 (H-2)	104.6 (C-1), 137.4 (C-4), 127.5 (C-5.9)	
		C-4	-	137.4	-	-	
		C-5,9	-	127.5	-	-	
		OCH_3	-	53.1	-	-	
N12	Protocatechuic acid	C-1	-	-	-		CV, CA, CI
		C-2	7.42 (m)	117.6	-	170.1 (CO), 152.4(C-4), 146.8 (C-3)	
		C-3	-	-	-		
		C-4	-	-	-		
		C-5	6.78 (d, *J*= 8.4 Hz)	115.5	-	152.4(C-4), 146.8 (C-3)	
		C-6	8.01 (dd, *J*= 8.4, 1.2 Hz)	130.6	-	170.1 (CO)	
		CO	-	170.1	-	-	
N13	Coumarin	C-2	-	170.2	-	-	CA, CI, CP-1, CP-2, CP-4
		C-3	-	117.3	-	-	
		C-4	-	145.6	-	-	
		C-5	7.63 (dd, *J*= 7.8, 1.2 Hz)	129.6	7.34 (H-6)	155.2(C-9), 145.6 (C-4), 133.1 (C-7), 117.3 (C-3), 120.3 (C-10)	
		C-6	7.34 (ddd, *J*= 8.4, 7.8, 1.2 Hz)	125.8	7.63 (H-5), 7.59 (H-7)	155.2 (C-9), 29.6 (C-5), 120.3 (C-10), 117.7 (C-6)	
		C-7	7.59 (ddd, *J*= 8.4, 6, 1.2 Hz)	133.1	7.34 (H-6),	155.2 (C-9),129.6 (C-5), 120.3 (C-10), 117.7 (C-8)	
		C-8	7.4 (dd, *J*= 6, 1.8 Hz)	117.7	-	170.2(C-2), 155.2 (C-9), 129.6 (C-5)	
		C-9	-	155.2	-	-	
		C-10	-	120.3	-	-	
N14	Vitamin B3 (Niacin)	C-2	9.02 (d, *J*=1.8 Hz)	-	-	-	CP-3

		C-4	8.28 (dt, *J*=1.8,7.8 Hz)	-	-	-	
		C-5	7.54 (dd, *J*=4.8,7.8 Hz)	-	-	-	
		C-6	8.69 (dd, *J*=1.2, 4.8 Hz)	-	-	-	
N15	Vitamin C (Ascorbic acid)	C-4	4.78	-	-	-	CP-3
		C-5	3.89 (t, *J*=6 Hz)	-	-	-	
		C-6	3.67 (d, *J*=6 Hz)	-	-	-	
N16	Vitamin (α-Tocopherol)	C-3	1.81 (m)	-	-	-	CP-3
		C-4	2.62 (t, *J*=6.6 Hz)	-	-	-	
		C-1′-C12′	1.1-1.6	-	-	-	
		C2-CH_3	1.24 (s)	-	-	-	
		C5-CH_3	2.07 (s)	-	-	-	
		C7-CH_3	1.98 (s)	-	-	-	
		C8-CH_3	1.95 (s)	-	-	-	
		C4′-CH_3	0.86 (d, *J*=6.6 Hz)	-	-	-	
		C8′-CH_3	0.85 (d, *J*=6.6 Hz)	-	-	-	
		C13′, C12′-CH_3	0.87 (d, *J*=6.6 Hz)	-	-	-	

Supplementary Figures

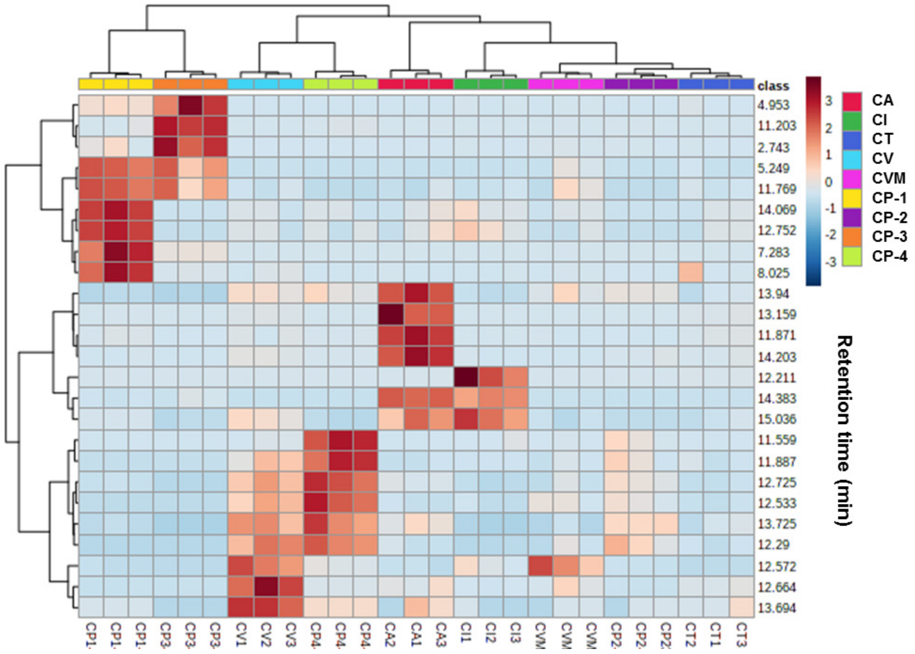

Figure S1: Heatmap analysis for all cinnamon samples based on SPME/GC-MS. The Y-axis represents the retention time (min) of the volatiles peak. The peak retention times for all samples and code are listed in **Table S1** and **Table 1**, respectively. Y-axis to the right shows retention times in min showing relative abundance among cinnamon specimens.

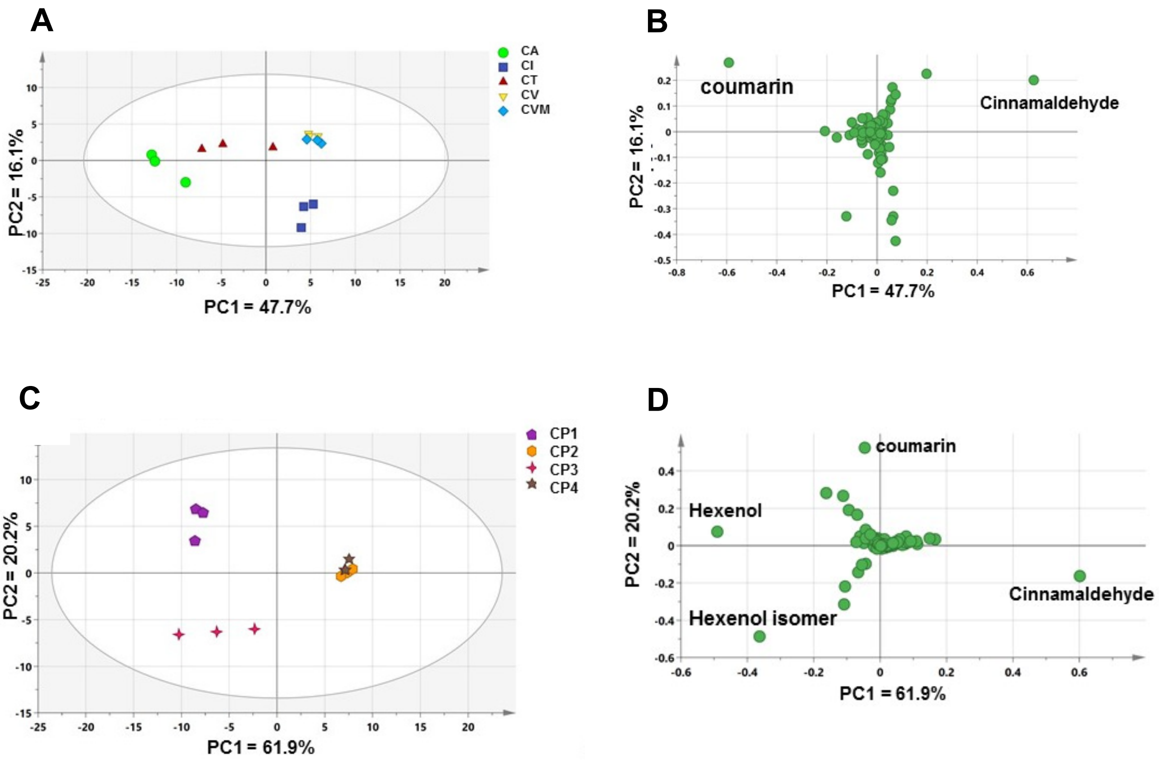

Figure S2: PCA score plot (**A** and **C**) and loading plot (**B** and **D**) for authenticated and commercial cinnamon samples, respectively, of cinnamon volatiles analyzed by SPME/GC-MS. The samples code is listed in **Table 1**.

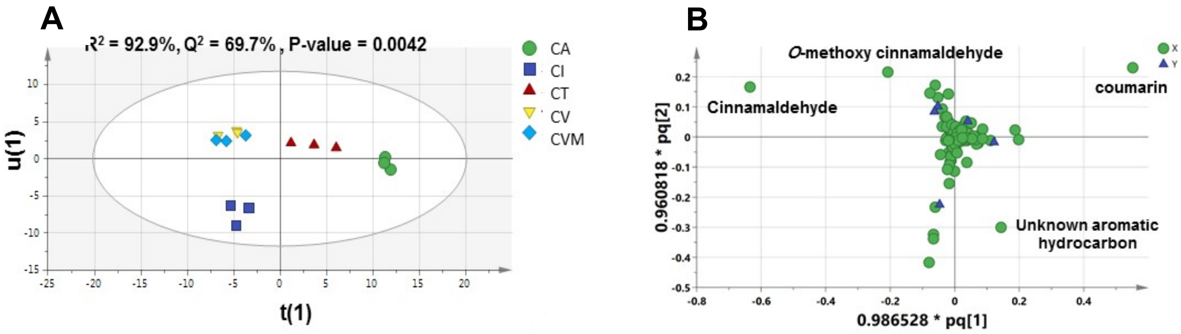

Figure S3: OPLS-DA score plot (**A**) and loading plot (**B**) of authenticated drugs for cinnamon volatiles analyzed by SPME/GC-MS. Samples code is listed in **Table 1**.

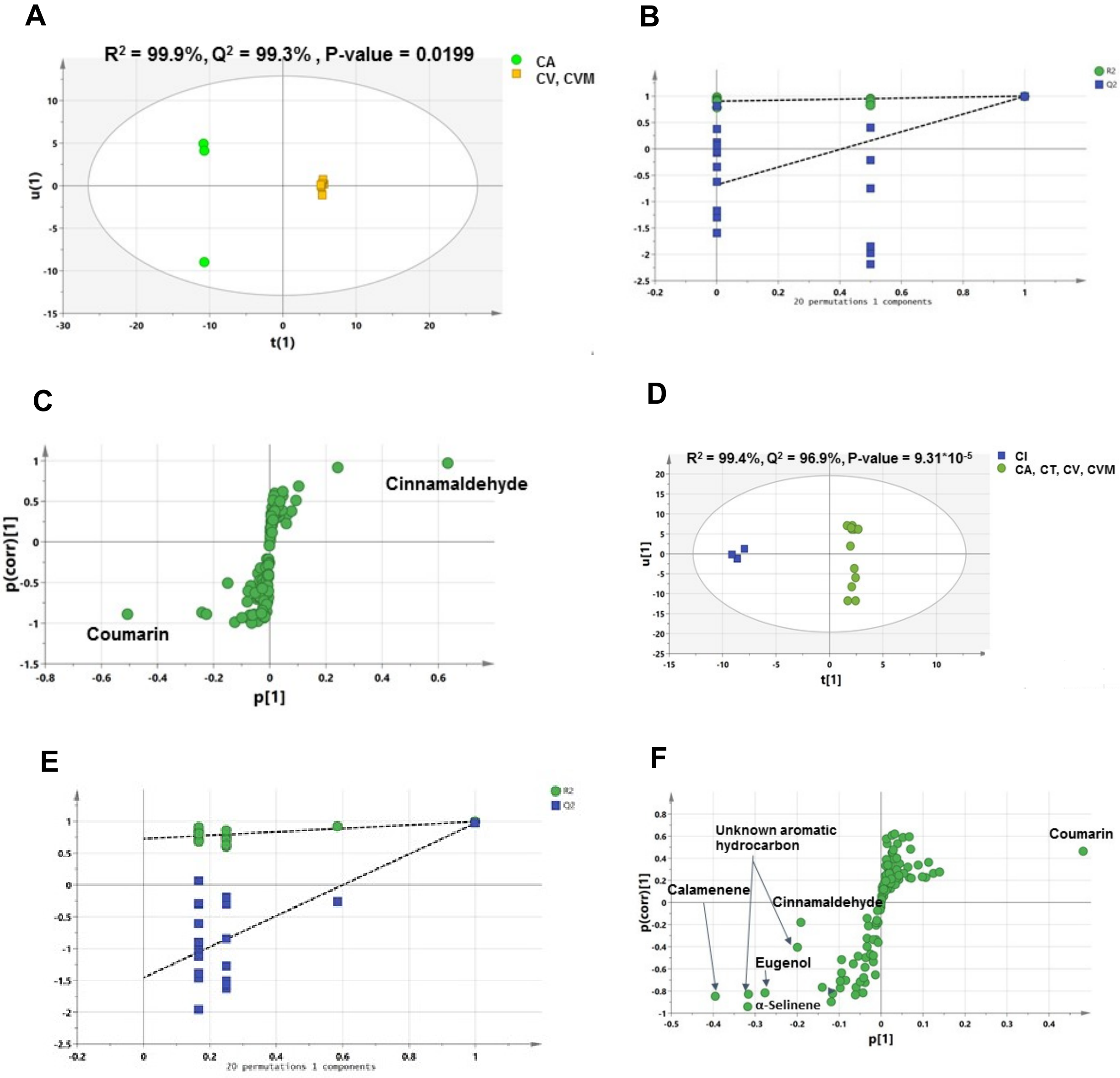

Figure S4: OPLS-DA for authentication of *Cinnamomum verum* against other *Cinnamomum* species for volatiles analyzed by SPME/GC-MS. **A)** and **D)** are score plot showing validation parameters, **B)** and **E)** are permutation plot, and **C)** and **F)** are S-plot for analysis CA and CI vs other authenticated cinnamon species. The samples code is listed in **Table 1**.

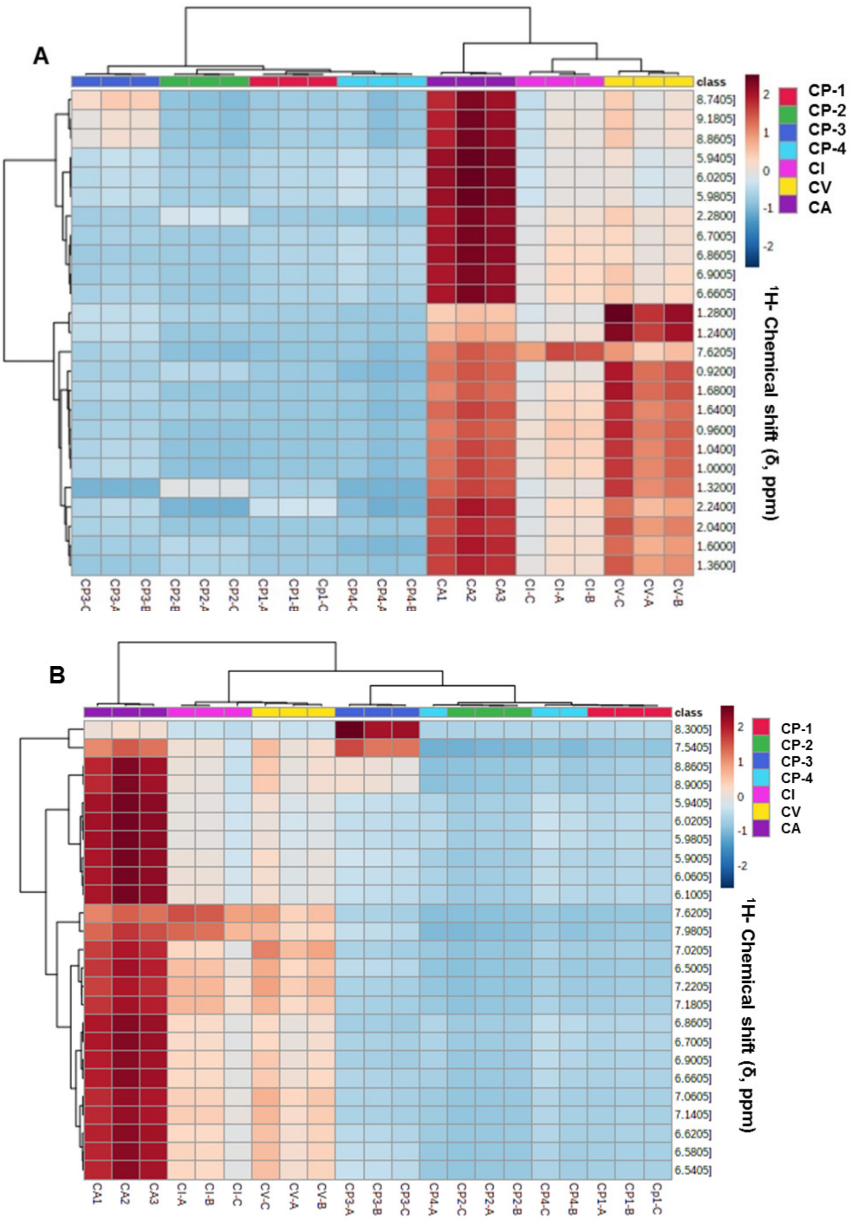

Figure S5: Unsupervised heatmap analysis of all cinnamon dataset. **A**) Full-scale (δ_H 0.0-10.0 ppm) and **B**) aromatic region (δ_H 5.5-10.0 ppm). The samples code is listed in **Table 1** and the metabolites assignment based on chemical shifts is summarized in **Table S2**. Y-axis to the right shows the chemical shifts showing relative abundance among cinnamon specimens.

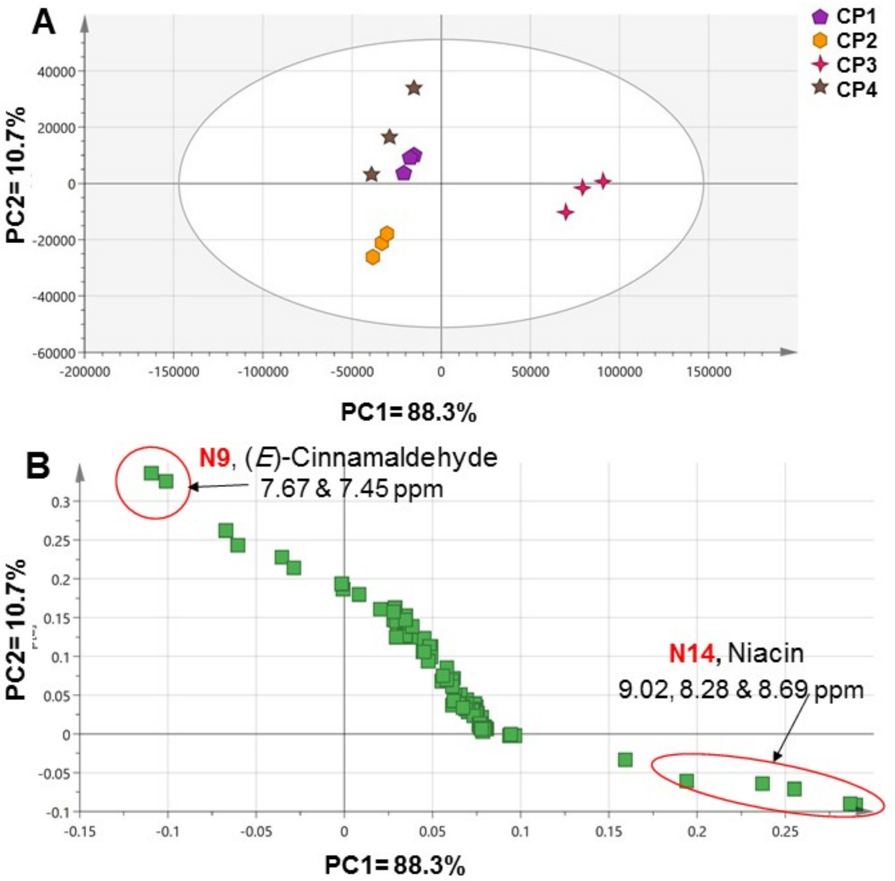

Figure S6: Unsupervised data analysis of the commercial cinnamon dataset in the aromatic region (δ_H 5.5-10.0 ppm). **A**) Principal component analysis (PCA) score plot, and **B**) PCA loading plot. The samples code is listed in **Table 1** and the metabolites assignment is summarized in **Table S2**.

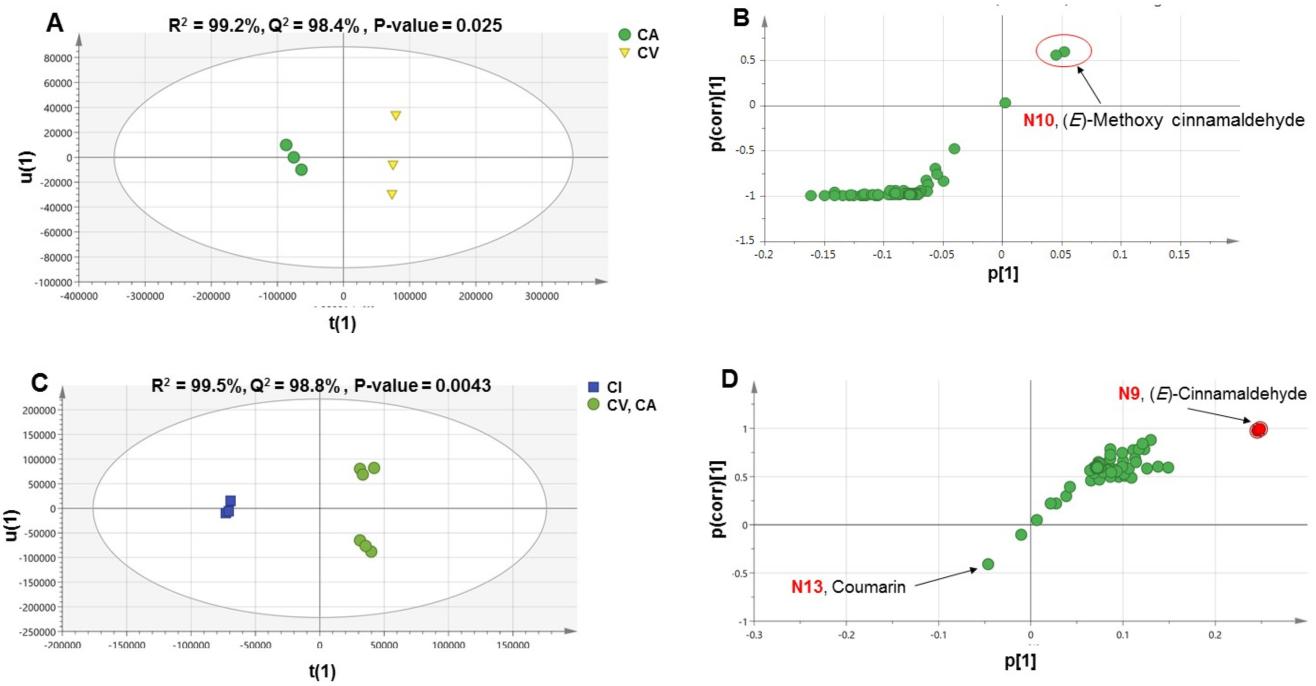

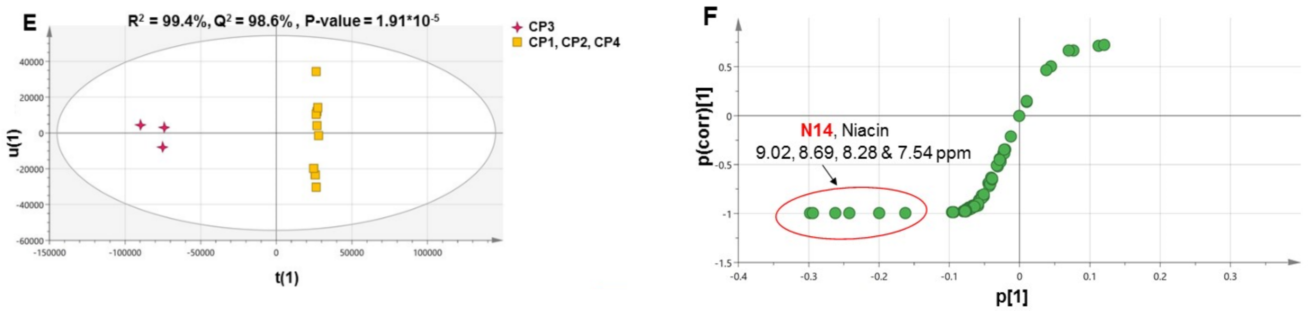

Figure S7: Supervised data analysis (OPLS-DA) of cinnamon dataset in the aromatic region (δ_H 5.5-10.0 ppm). OPLS score plot (**A, C,** and **E**) and loading plot **(B, D,** and **F**) for modelling of CA vs CV, CI vs CV and CA, in addition to CP3 vs CP1, CP2 and CP4. The samples code is listed in **Table 1** and the metabolites assignment is summarized in **Table S2**.

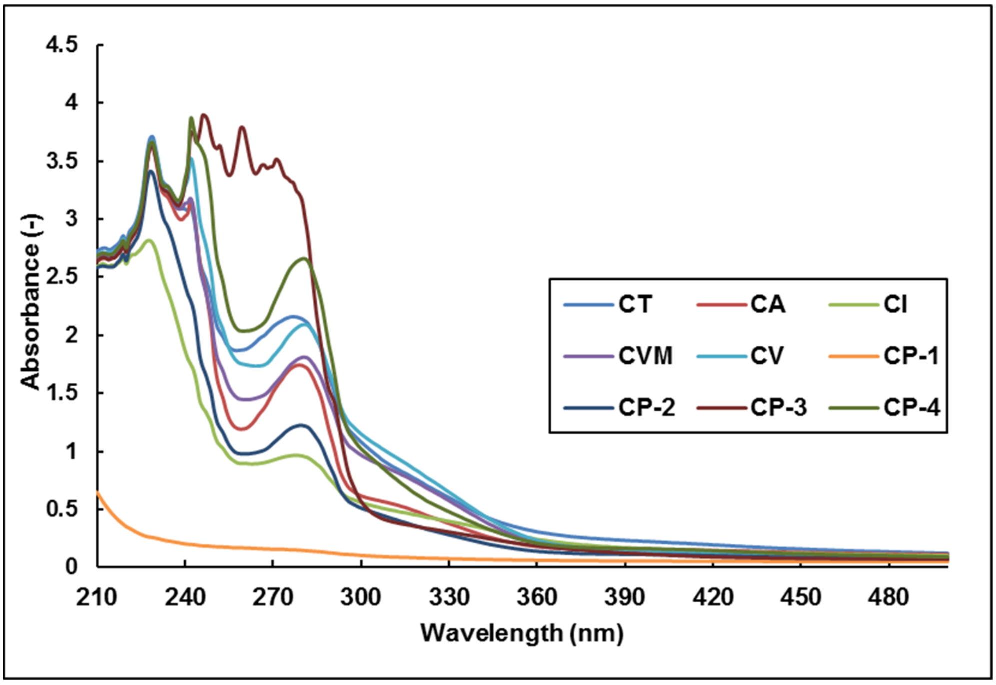

Figure S8: UV/Vis spectra of investigated authenticated and commercial cinnamon samples. The samples code is listed in **Table 1**. The figure shows the unique spectrum of CP-3.

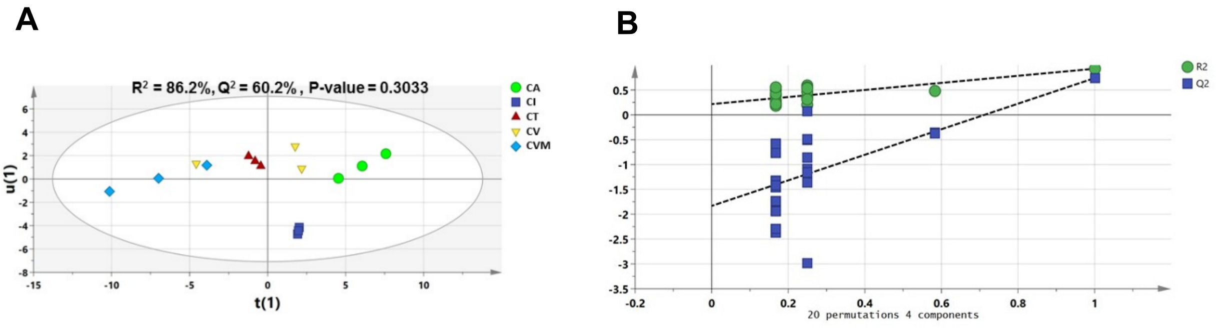

Figure S9: Supervised OPLS-DA score plot (**A**) of authenticated cinnamon samples and permutation calculation (**B**). The samples code is listed in **Table 1**.

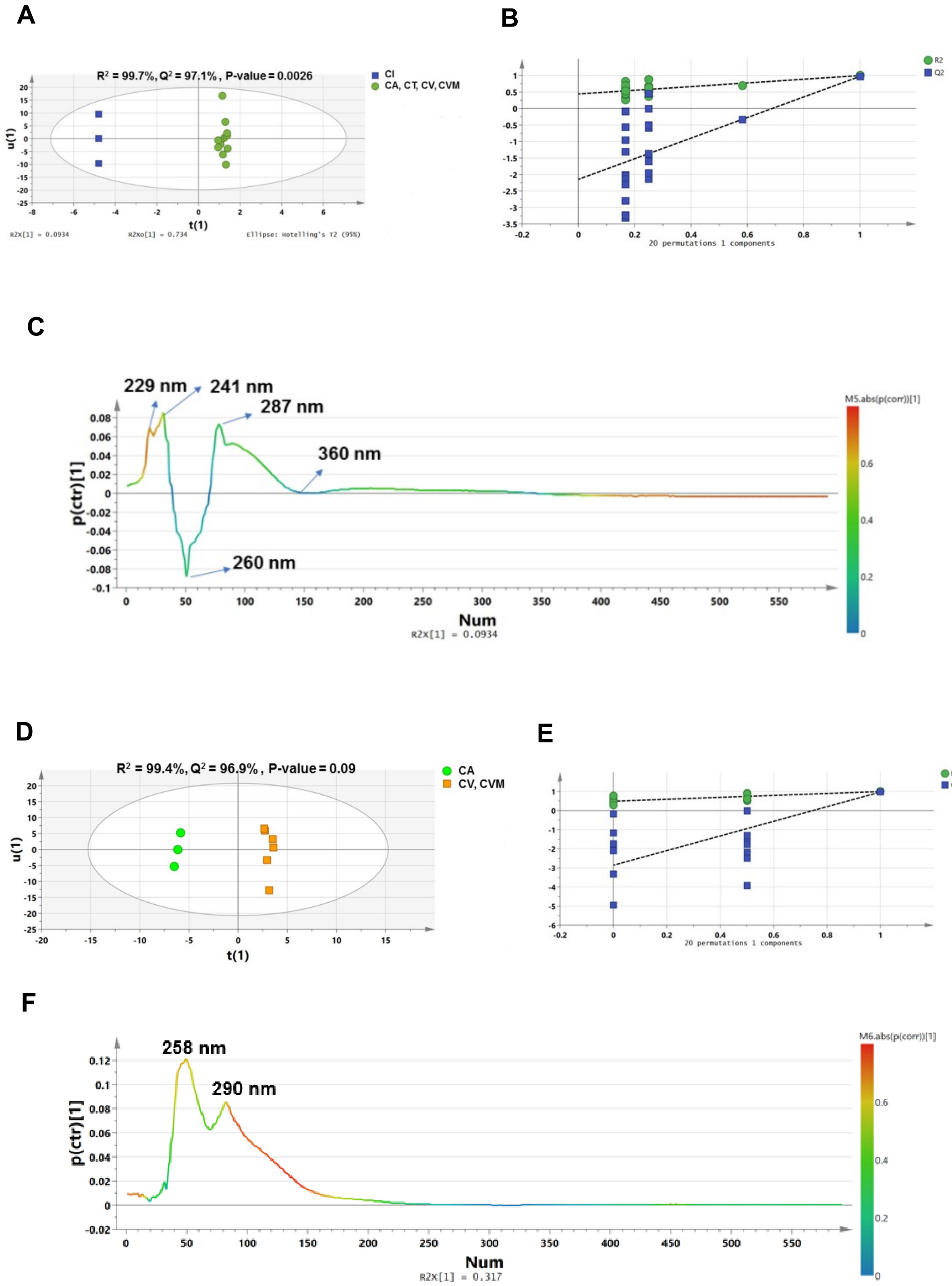

Figure S10: Supervised OPLS-DA score plot (**A**) of CI against other authenticated cinnamon samples and permutation results (**B**), OPLS-DA line-plot (**C**). OPLS-DA score plot of CA against CV and CVM samples (**D**), permutation results (**E**), and OPLS-DA line-plot (**F**). The samples code is listed in **Table 1**.

Raw data
A sample (CP-1) of the NMR data can be shared under the following links

- ^{1}H-NMR:
  https://drive.google.com/drive/u/0/folders/1W306Qy6wylD1IyJU2hzBQSsklrHQutD0
- COSY:
  https://drive.google.com/drive/u/0/folders/1HAqf0nNod3yh49bRlssnfuoirYTyl-Xw
- HMBC:
- https://drive.google.com/drive/u/0/folders/1zJ93i9_L4XzWi-kEEAhl4A_BAKgilvGo
- HSQC:
  https://drive.google.com/drive/u/0/folders/12pyeOxEITW09VTWQlrHr9BKKFG3qf3ag

  NMR binning matrix:
  https://pharmtantaedu-my.sharepoint.com/:x:/g/personal/ahmed_zayed1_pharm_tanta_edu_eg/EbNs13uJq3RJuvWCoDTCF2wB5IMBJVjpMNRZYgPDoEHbow?e=qkkAtY

Also, the raw data of SPME/GC-MS can be found in this link

- SPME/GC-MS identification sheet:

https://pharmtantaedu-my.sharepoint.com/:x:/g/personal/ahmed_zayed1_pharm_tanta_edu_eg/EUMD6pWtVOtAtjJjxk937iYBSDM9ngUa0pXV8oqar2zHag?e=KUcTLf

- Raw data net cdf universal files:

1. CV:

https://pharmtantaedu-my.sharepoint.com/:u:/g/personal/ahmed_zayed1_pharm_tanta_edu_eg/EbjzfSUsLAhEtESTAA3GxPsB2-bRTKh1GC5tEAORfSiwSA?e=mYhOhn

2. CVM:

https://pharmtantaedu-my.sharepoint.com/:u:/g/personal/ahmed_zayed1_pharm_tanta_edu_eg/EYtPHIaoBQVNnRTiWbEDfr4BNG4i4nvlV_KkWRz_DmiIkw?e=0Wmd5o

3. CT:

https://pharmtantaedu-my.sharepoint.com/:u:/g/personal/ahmed_zayed1_pharm_tanta_edu_eg/ERwjumiSE7JLt-7npAW_aC4BuMlfigs6ho6KXnb7tgc7-w?e=hHNalj
